# ﻿Fishes (Actinopterygii) of the rapids and associated environments in the lower Vaupés River Basin: an undiscovered Colombian Amazon diversity

**DOI:** 10.3897/zookeys.1203.100642

**Published:** 2024-05-30

**Authors:** Alexander Urbano-Bonilla, Jorge E. Garcia-Melo, Mateo Esteban Peña-Bermudez, Omar Eduardo Melo-Ortiz, Oscar Stiven Ordoñez, Sandra Bibiana Correa, Tiago P. Carvalho, Javier A. Maldonado-Ocampo

**Affiliations:** 1 Laboratorio de Ictiología, Unidad de Ecología y Sistemática (UNESIS), Departamento de Biología, Facultad de Ciencias, Pontificia Universidad Javeriana, Carrera 7 N° 43-82, Bogotá, 110231, D.C., Colombia; 2 Universidad de Ibagué, Facultad de Ciencias Naturales y Matemáticas, Programa de Biología Ambiental, Tolima-Colombia, Ibague, Colombia; 3 Department of Wildlife, Fisheries and Aquaculture, Mississippi State University, Mississippi, USA; † Deceased

**Keywords:** Conservation, freshwater, Neotropical fishes, new records, PhotaFish System, range expansion, taxonomy

## Abstract

The Vaupés River stands out as one of the few within the Amazon basin due to its numerous rapids. These riverine fast-flowing sections not only provide habitat to highly specialized fishes but also function as natural barriers hindering the movement of fish along its course. During a fish-collecting expedition in the lower Vaupés River basin in Colombia, 95 species were registered belonging to 30 families and seven orders. Despite recent inventories in the region, our comprehensive sampling efforts particularly focused on the rapids and associated rheophilic fauna, allowing us to contribute the first records of four fish species in Colombia (*Mylopluslucienae* Andrade, Ota, Bastos & Jégu, 2016, *Tometesmakue* Jégu, Santos & Jégu, 2002, also first record of the genus, *Leptodoraspraelongus* (Myers & Weitzman, 1956), and *Eigenmanniamatintapereira* Peixoto, Dutra & Wosiacki, 2015) and six presumably undescribed species (i.e., *Jupiaba* sp., *Moenkhausia* sp., *Phenacogaster* sp., *Bunocephalus* sp., *Hemiancistrus* sp., and *Archolaemus* sp.). In this study, a commented list of the ichthyofauna of these environments is presented, as well as a photographic catalog of fish species integrated into the CaVFish Project – Colombia.

## ﻿Introduction

The Neotropical Region is the biogeographic region with the highest number of freshwater fish species globally, and recent estimates suggest approximately 9,000 species (Reis et al. 2016; Birindelli and Sidlauskas 2018; Dagosta and de Pinna 2019). Some localities in the Amazon River basin often exhibit remarkably high fish species richness that surpasses the hundreds (Albert and Reis 2011). The extraordinary radiation of fishes that occurred in the Neotropical region is often explained as the product of geographic events over extended geological periods (Albert and Crampton 2010; Albert and Reis 2011; Albert et al. 2020), but also lineage diversification related to habitat utilization and trophic specialization (Lujan et al. 2012; Lujan and Conway 2015; Arbour and López-Fernández 2016; Roxo et al. 2017; Kolmann et al. 2021).The Vaupés River and its fish have a long history of expeditions that began in the 18^th^ and 19^th^ centuries by early naturalists, such as Alexandre Rodrigues Ferreira, Alexander Von Humboldt, and Alfred Russel Wallace (Lima et al. 2005). Recent analyses of fish biodiversity and hotspots in Amazonia suggested that the Vaupés hydrographic basin in its entirety has high values of species richness and endemism (Jézéquel et al. 2020a), species with high levels of irreplaceability, representativeness, and degree of vulnerability (Jézéquel et al. 2020b). Unlike other tributaries of the Amazon, the numerous rapids of the Vaupés River serve as habitat and provide food (e.g., Podostemaceae aquatic plants) for fish (Lima et al. 2005); in addition, rapids act as natural barriers that affect the dispersal of some fish and harbor rheophilic and endemic fish species (Londoño-Burbano and Urbano-Bonilla 2018; Urbano-Bonilla et al. 2023).

The River Negro Basin, of which Vaupés River is a major tributary, has a rich ichthyofauna, with 1,165 species known to science. A large portion of these species are shared with adjacent basins (i.e., Orinoco), but ~ 15% are endemic (156 species; Beltrão et al. 2019). The western Rio Negro tributaries are known for their distinctive rheophilic fish fauna (Lima et al. 2005). A total of 224 fish species are known to occur in the Vaupés River in Colombia (Bogotá-Gregory et al. 2020, 2022a); some of these species are endemic to this basin, while others are widely distributed in the Amazon basin and adjacent basins such as the Orinoco and those within the Guiana Shield (van der Sleen and Albert 2017; Beltrão et al. 2019; Bogotá-Gregory et al. 2020, 2022a, 2022b; Taphorn et al. 2022).

Despite historic and contemporary sampling efforts, the Vaupés River remains largely under-sampled mainly because of its remote geographical location and numerous rapids, preventing access and navigation. Also, after putting an end to a 60-year conflict between the Colombian state and one of the oldest guerrilla organizations in the world (Fuerzas Armadas Revolucionarias de Colombia-Ejército del Pueblo FARC-EP), biological expeditions were carried out filling an important information gap relative to this previously unreachable area (Botero 2020; Irwin 2023). Recent studies evaluating the sampling efforts to inventory Amazon River basin ichthyofauna reveal that extensive areas in southwestern Colombia remain almost unsampled (Jézéquel et al. 2020a). Recent reviews purported new records for the basin and Colombia suggesting that the biodiversity knowledge of the area is still incipient (Bogotá-Gregory et al. 2020, 2022a).

**Figure 1.**
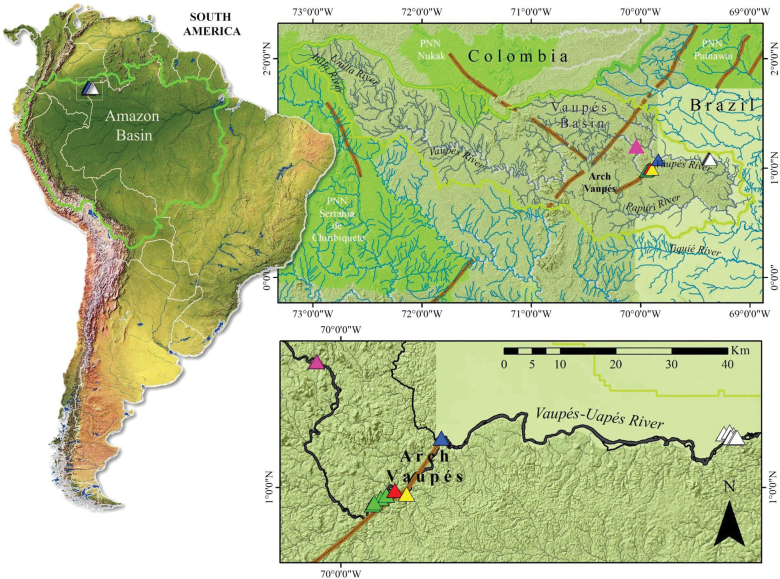
Location of the Vaupés River in Colombia and distribution of sampling sites along the lower Vaupés River. Key: pink triangle – Trubón Community Rapids; green triangle – Villa Fátima Community Rapids; red triangle – Nana Community Rapids; yellow triangle – Macucú Community Rapids; white triangle – Matapí Community Rapids, and blue triangle – military base.

Here we describe the results of an expedition to the lower Vaupés River basin with the goal of investigating fish species associated with the rapids and surrounding environments in the Vaupés arc (Miocene ≈ 10 Mya; see Fig. 1). Tragically during this expedition Colombian, in the Matapí Rapids in the Vaupés River, the boat transporting researchers capsized, and the leader of the expedition, ichthyologist Javier Maldonado-Ocampo passed away (read more in Urbano-Bonilla et al. 2021). This document is a tribute to the effort of Javier, who dedicated his life to the generation and transmission of knowledge aimed at recognizing the diversity of Colombian fishes and rescuing ancestral knowledge.

## ﻿Materials and methods

### ﻿Study area and site characterization

This study was carried out in the lower Vaupés River basin in Colombia, more specifically in the Municipality of Mitú, Department of Vaupés. The fish collections were conducted within the indigenous communities of Trubón, Villa Fátima, Nana, Macucú, and Matapí (Fig. 1). We characterized the river channel depth profile from shore to shore. First, we measured the river width with a laser Rangefinder (Nikon Forestry Pro) and divided the river into 5–10 segments. We conducted readings at each location by driving the boat across the river while reading depth on a Hummingbird water depth sonde (model Fishfinder 525) connected to a transducer mounted on an external pole that was carried on the side of the boat. Distance was tracked with a GPS unit (Garmin 76CSx). We measured water transparency with a Secci disk. Temperature and dissolved oxygen was measured at the water surface (YSI Pro 20).

### ﻿Sampling methods

We sampled along a stretch of ~ 140 km of the main river course. Fish collection follows animal care guidelines provided by the American Society of Ichthyologists and Herpetologists 2013 (https://www.asih.org/resources).

**Figure 2.**
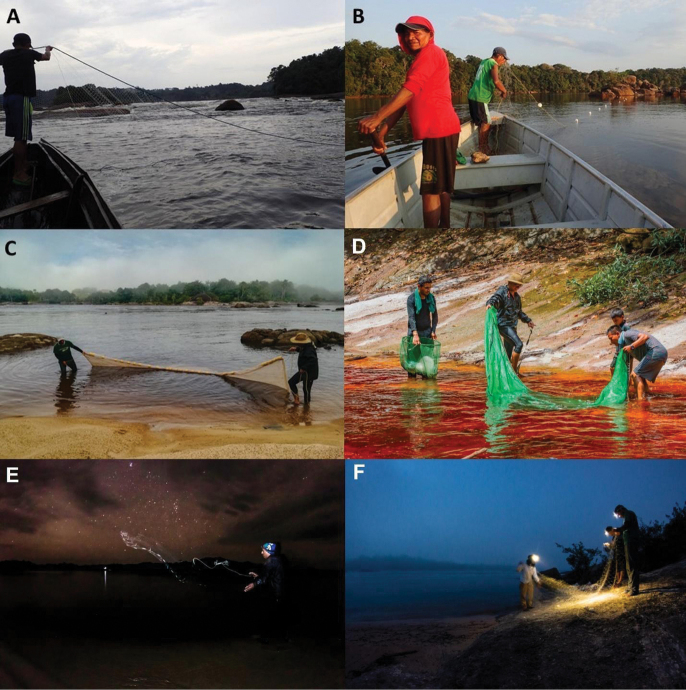
Gear and habitats sampled in the lower Vaupés River, Mitú, Colombia. Gillnets deployed on **A** rapids and **B** beaches below rapids (photographs by SBC). Seine-net used on the beaches of the **C** rapids and **D** associated stream; night fishing with **E** cast net and **F** gillnets (photographs by JEG-M).

Collections were conducted during the low water period (from February 21 to March 3, 2019), in which we carried standardized sampling with different fishing gear in rapids and surrounding habitats. Four monofilament nylon gillnets: two multi-panel gill nets, 25 m long × 2.5 m depth with five equal length panels of different mesh sizes (2.54, 3.81, 5.08, 6.35, and 7.62 cm stretched mesh size); one 50 m long and one 100 m long, both 14.7 cm stretched mesh size, were deployed at rapids, shallow areas of the main channel, and beaches directly below rapids for 3 hours (morning and night; 6 hrs total per day). Beaches were additionally sampled by five passes with beach seines (3 m long, 2 m high, and 0.5 cm mesh size) and ten cast net throws. Five passes were made with a seine net (3 m long, 2 m high, and 0.5 cm mesh) in streams surrounding the rapids during the day (Fig. 2). This sampling was coupled with 1.5 hours of nocturnal collections with dip nets. Opportunistic sampling was conducted by snorkeling and dip netting in shallow areas.

### ﻿Photo and CaVFish Project - Colombia database

Each species was photographed alive in the field using the PhotaFish System (García-Melo et al. 2019) in white and black backgrounds. Subsequently, the images were processed assigning the taxonomy established in the laboratory or by direct visual inspection when the voucher was not available. Likewise, tagging and editing were performed using the pipeline developed by the CaVFish Project - Colombia (https://cavfish.unibague.edu.co/). A few species not photographed in the field were photographed in the laboratory following similar protocols.

### ﻿Specimen preservation

The collected specimens were euthanized by overdose with clove oil (*Syzygiumaromaticum* (L.) Merr. y Perry, 1939, 0.3 ml/0.25L; Lucena et al. 2013). Fishes were fixed in 10% formaldehyde and later preserved in 70% ethanol for storage. Before formalin fixation in the field, we conducted tissue sampling on euthanized specimens, preserving muscles or fin clips in 2 ml vials containing 96% ethanol. Identification followed taxonomic keys for genus-level assignment (van der Sleen and Albert 2017), specialized literature for species-level identification, and comparison with reference material deposited in the Javeriano Museum of Natural History “Lorenzo Uribe Uribe S.J” (**MPUJ**) collection. Databases of this study are available at https://ipt.biodiversidad.co/sib/resource?r=peces_del_rio_vaupes. Additionally, photographs of live specimens were sent to taxonomic experts for verification and identification (see acknowledgments section). The classification system follows Fricke et al. (2023) and Dornburg and Near (2021) within which fish orders, families, genera, and species were listed alphabetically.

Unfortunately, a small portion of these fishes were lost during the expedition and therefore were represented only by photographs (or pictures + tissue samples) and are not associated with vouchered specimens.

## ﻿Results

### ﻿Sampling stations and water physicochemical characteristics

In the lower Vaupes River, in total, we sampled 16 sites (Figs 1, 2, Table 1). The drainage network includes streams, lagoons, river beaches, and river rapids ranging from 2–3 m wide up to 380 m in the main channel. In the latter, the depth varied from a few centimeters at the shore to 18 m. The water is dark in color (brown-black) with relatively high transparency (assessed by Secchi disk, mean and standard deviation ranging between 108 to 122.50 ± 10.61 cm). Temperature ranged between 28.15 ± 0.21 and 29.65 ± 0.21 °C, and surface dissolved oxygen between 6.41 ± 0.01 and 7.63 ± 0.25 mg/L.

**Table 1.** Description of sampled sites in the lower Vaupés River, Amazon basin, Colombia. Coordinates in degrees, minutes, seconds’ format, and altitude in meters above sea level. Localities are ordered by altitude.

Locality description	GPS coordinates	Altitude
Sandy beach on Vaupés River at Resguardo Trubón	1°12'8"N, 70°2'20"W	164
Caño Danta creek tributary to Vaupés River near Villa Fátima	0°58'57"N, 69°56'9"W	168
Vaupés river rapids area, in front of Villa Fátima	0°58'21"N, 69°56'58"W	150
Sandy beach on Vaupés River at Villa Fátima	0°58'33"N, 69°56'47"W	148
Sandy beach and rocky shore on Vaupés River downstream Villa Fátima	0°59'16"N, 69°55'36"W	148
Vaupés River at rapids in front of community of Naná	0°59'44"N, 69°54'48"W	147
Macucú Rapids and sandy beach at community of Macucú	0°59'22"N, 69°53'39"W	144
Vaupés River near Militar Base	1°4'46"N, 69°50'18"W	144
Downstream of the rapids of Caño Almidón, tributary to Vaupés River, upstream of community of Matapí	1°5'11"N, 69°23'1"W	150
Creek tributary to Vaupés River near community of Matapí	1°5'5"N, 69°22'5"W	146
Sandy beach at Vaupés River upstream cachivera Tapira Geral near community of Matapí	1°5'21"N, 69°22'27"W	138
Sandy beach at community of Matapí	1°4'49"N, 69°21'50"W	134
Laguna Arcoiris, small lagoon adjacent to Vaupés River at community of Matapí	1°4'48"N, 69°22'23"W	133
Sandy beach ~ 300 m downstream cachivera Tapira Geral near community of Matapí	1°4'49"N, 69°22'20"W	133
Caño Colibrí, near community of Matapí	1°4'47"N, 69°21'54"W	132
Sandy beach and rocky shore on Vaupés River River at community of Matapí	1°4'49"N, 69°21'45"W	129

### ﻿Composition

We collected 95 species (Tables 2, 3), 85 of those identified at the species level and ten at the genus level. These species are distributed in 30 families and seven orders. The orders Characiformes (54 spp.) and Siluriformes (21 spp.) represent more than 78% of the total diversity of fish; the remaining orders have between five and nine species (Table 2). In addition, 44 new records are added to the previous list of fishes from the Vaupés River basin of Bogotá-Gregory et al. (2022a).

**Table 2.** Number and percentage of families, genera, and species per order.

Order	Family	%	Genus	%	Species	%
Characiformes	15	50	30	46.88	54	56.84
Siluriformes	8	26.67	19	29.69	21	22.11
Bleniiformes	3	10	7	10.94	10	10.53
Gymnotiformes	1	3.33	4	6.25	4	4.21
Acanthuriformes	1	3.33	2	3.13	4	4.21
Gobiiformes	1	3.33	1	1.56	1	1.05
Synbranchiformes	1	3.33	1	1.56	1	1.05
	**30**	**100**	**64**	**100**	**95**	**100**

**Table 3.** List of fish species collected in the lower Vaupés River and their respective voucher numbers at MPUJ, figure numbers, and literature that support taxonomic identification. Species with ^1^ represent new records for Colombia; ^2^ represents putative new species; and ^3^ represents records not included in Bogotá-Gregory et al. (2022a).

ORDER/Family/Species	Voucher	fig.	Literature
**CHARACIFORMES**			
**Acestrorhynchidae**			
*Acestrorhynchusmicrolepis* (Jardine, 1841)	uncatalogued, photo voucher only	Suppl. material 1: fig. S1	López-Fernández and Winemiller 2003
**Anostomidae**			
*Gnathodolusbidens* Myers, 1927	MPUJ 14496	Suppl. material 1: fig. S2	Mendes and Jégu 1987
*Leporinusbrunneus* Myers, 1950	MPUJ 14504, 14507	Suppl. material 1: fig. S3	Chernoff et al. 1991
*Leporinusfasciatus* (Bloch, 1794)	MPUJ 14369, 14478	Suppl. material 1: fig. S4	Taphorn 2003
*Leporinusniceforoi* Fowler, 1943 ^3^	MPUJ 14476, 14539	Suppl. material 1: fig. S5	Sidlauskas et al. 2011
*Leporinusyophorus* Eigenmann, 1922 ^3^	MPUJ 14506	Suppl. material 1: fig. S6	Taphorn 2003
**Bryconidae**			
*Bryconpesu* Müller & Troschel, 1845	MPUJ 14382, 14389, 14405, 14449, 14472, 14473, 14516, 14517, 14531, 14383	Suppl. material 1: fig. S7	Lima 2017
**Characidae**			
*Bryconamericusorinocoensis* Román-Valencia, 2003	MPUJ 14379, 14386, 14423, 14438, 16524	Suppl. material 1: fig. S8	Román-Valencia 2003
*Creagrutusmaxillaris* (Myers, 1927)	MPUJ 14388, 14428, 14429, 14430, 14534	Suppl. material 1: fig. S9	Vari and Harold 2001
*Creagrutusvexillapinnus* Vari & Harold, 2001 ^3^	MPUJ 14394, 14413, 14434	Suppl. material 1: fig. S10	Vari and Harold 2001
*Hemigrammusanalis* Durbin, 1909	MPUJ 14480, 14486	Suppl. material 1: fig. S11	Géry 1977
*Hemigrammusbellottii* (Steindachner, 1882)	MPUJ 14455, 14456, 14484, 14491, 14546	Suppl. material 1: fig. S12	Géry 1977
*Hemigrammusgeisleri* Zarske & Géry, 2007 ^3^	MPUJ 14421, 14540, 16520	Suppl. material 1: fig. S13	Zarske and Géry 2007
*Hemigrammusluelingi* Géry, 1964	MPUJ 14545	Suppl. material 1: fig. S14	Géry 1977
*Jupiabaanteroides* (Géry, 1965)	MPUJ 14487	Suppl. material 1: fig. S15	Zanata 1997; Ferreira et al. 2009
*Jupiabascologaster* (Weitzman & Vari, 1986) ^3^	MPUJ 14436, 16515	Suppl. material 1: fig. S16	Zanata 1997; Ferreira et al. 2009
*Jupiabazonata* (Eigenmann, 1908)	MPUJ 14435	Suppl. material 1: fig. S17	Zanata 1997; Ferreira et al. 2009
*Jupiaba* sp. ^2^	MPUJ 14385, 14424, 14440, 14446, 14467, 14475, 14488, 14538, 14370	Suppl. material 1: fig. 4A	Zanata 1997; Ferreira et al. 2009
*Knodus* sp. 1 ^3^	MPUJ 14447	Suppl. material 1: fig. S19	Van der Sleen et al. 2018
*Knodus* sp. 2 ^3^	MPUJ 14536	Suppl. material 1: fig. S20	Van der Sleen et al. 2018
*Knodus* sp. 3	MPUJ 14452, 14407	Suppl. material 1: fig. S21	Van der Sleen et al. 2018
*Microschemobryconcallops* Böhlke, 1953	MPUJ 14533	Suppl. material 1: fig. S22	Lima et al. 2013
*Microschemobryconcasiquiare* Böhlke, 1953	MPUJ 14422, 14448, 16521	Suppl. material 1: fig. S23	Lima et al. 2013
*Moenkhausiabrowni*^3^ Eigenmann, 1909	MPUJ 14397, 16514, 16517	Suppl. material 1: fig. S24	Géry 1977
*Moenkhausiaceros* Eigenmann, 1908	MPUJ 14366, 14541	Suppl. material 1: fig. S25	Géry 1977
*Moenkhausiacollettii* (Steindachner, 1882)	MPUJ 14457, 14460, 14492, 14537, 14544	Suppl. material 1: fig. S26	Géry 1977
*Moenkhausiacotinho* Eigenmann, 1908	MPUJ 14494	Suppl. material 1: fig. S27	Mathubara and Toledo-Piza 2020
*Moenkhausialata* Eigenmann, 1908	MPUJ 14432	Suppl. material 1: fig. S29	Marinho and Langeani 2016
*Moenkhausiamelogramma*^3^ Eigenmann, 1903	MPUJ 14543, 14367, 14410, 14437	Suppl. material 1: fig. S30	Soares et al. 2020
*Moenkhausiamikia* Marinho & Langeani, 2010	MPUJ 14371, 14400, 14414, 14419, 14453, 14489, 14439	Suppl. material 1: fig. S31	Marinho and Langeani 2016
*Moenkhausia* sp. ^2^	MPUJ 14427, 14542, 14374, 14411, 14443	Suppl. material 1: fig. 4C	Marinho and Langeani 2016
*Phenacogaster* sp. 1	MPUJ 14373, 14425	Suppl. material 1: fig. S32	Lucena and Malabarba 2010
*Phenacogaster* sp. 2 ^2^	MPUJ 14390, 14364	Suppl. material 1: fig. 4B	Lucena and Malabarba 2010
*Tetragonopteruschalceus* Spix & Agassiz, 1829	MPUJ 14483	Suppl. material 1: fig. S34	Silva et al. 2016
**Chilodontidae**			
*Caenotropuslabyrinthicus* (Kner, 1858) ^3^	MPUJ 14409, 14477, 16516	Suppl. material 1: fig. S35	Vari et al. 1995
**Crenuchidae**			
*Characidiumdeclivirostre* Steindachner, 1915 ^3^	MPUJ 14497	Suppl. material 1: fig. S36	Armbruster et al. 2021
*Characidiumlongum* Taphorn, Montana & Buckup, 2006	MPUJ 14365	Suppl. material 1: fig. S37	Taphorn et al. 2006
*Characidiumpteroides* Eigenmann, 1909 ^3^	MPUJ 14384	Suppl. material 1: fig. S38	Taphorn et al. 2006
**Ctenolucidae**			
*Boulengerellamaculata* (Valenciennes, 1850)	MPUJ 14502	Suppl. material 1: fig. S39	Vari 1995
**Curimatidae**			
*Cyphocharaxleucostictus* (Eigenmann & Eigenmann, 1889)	MPUJ 14376, 14399, 14418	Suppl. material 1: fig. S40	Vari 1992
*Cyphocharaxspilurus* (Gunther,1864)	MPUJ 14368, 14391	Suppl. material 1: fig. S41	Vari 1992
**Cynodontidae**			
*Hydrolycuswallacei* Toledo-Piza, Menezes & Santos, 1999	MPUJ 14547	Suppl. material 1: fig. S42	Toledo-Piza et al. 1999
**Gasteropelecidae**			
*Carnegiellastrigata* (Gunther, 1864)	MPUJ 14493	Suppl. material 1: fig. S43	Weitzman 1960
**Hemiodontidae**			
*Argonecteslongiceps* (Kner, 1858)	MPUJ 14554, 16519	Suppl. material 1: fig. S44	Langeani 2018
*Bivibranchiafowleri* (Steindachner, 1908)	MPUJ 14403, 14426, 14442, 14535, 14416	Suppl. material 1: fig. S45	Langeani 2018
*Hemiodusthayeria* Böhlke, 1955	MPUJ 14377, 14444, 14514	Suppl. material 1: fig. S46	Langeani 2018
**Iguanodectinae**			
*Bryconopsgiacopinii* (Fernández -Yépez, 1950)	MPUJ 14462, 14463	Suppl. material 1: fig. S47	Chernoff and Machado-Alisson 2005
*Bryconopscollettei* Chernoff & Machado-Alisson, 2005 ^3^	MPUJ 14461, 14464, 16523	Suppl. material 1: fig. S48	Chernoff and Machado-Alisson 2005
**Lebiasinidae**			
*Copellanattereri* (Steindachner, 1876)	MPUJ 14548	Suppl. material 1: fig. S49	Marinho and Menezes 2017
**Serrasalmidae**			
*Mylopluslucienae* Andrade, Ota, Bastos, 2016 ^1^	MPUJ 14524, 14525, 14528	Suppl. material 1: fig. 3A	Andrade et al. 2016
*Serrasalmusstriolatus* Steindachner, 1908^3^	Uncatalogued, photo voucher only	Suppl. material 1: fig. S51	Taphorn 2003
*Serrasalmusmanueli* (Fernández-Yépez & Ramírez, 1967)	MPUJ 14417	Suppl. material 1: fig. S52	Taphorn 2003
*Tometesmakue* Jégu, Santos & Jégu, 2002 ^1^	MPUJ 14498, 14527, 14529, 14550, 14553	Suppl. material 1: fig. 3B	Jégu et al. 2002
**Triportheidae**			
*Triportheusalbus* Cope, 1872	MPUJ 16522	Suppl. material 1: fig. S54	Malabarba 2004
**SILURIFORMES**			
**Aspredinidae**			
*Bunocephalus* sp.^2^	MPUJ 14433	Suppl. material 1: fig. 4B	Carvalho et al. 2018
**Auchenipteridae**			
*Ageneiosusinermis* (Linnaeus, 1766)	MPUJ 14515	Suppl. material 1: fig. S56	Ribeiro et al. 2017
*Tatiaintermedia* (Steindachner, 1877)	Uncatalogued, photo voucher only	Suppl. material 1: fig. S57	Sarmento-Soares and Martins-Pinheiro 2008
**Doradidae**			
*Amblydorasaffinis* Kner, 1855	MPUJ 14398	Suppl. material 1: fig. S58	Birindelli and de Souza 2018
*Centrodorashasemani* (Steindachner, 1915) ^3^	Uncatalogued, photo voucher only	Suppl. material 1: fig. S59	Birindelli and de Souza 2018
*Dorasphlyzakion* Sabaj Pérez & Birindelli, 2008 ^3^	MPUJ 14521, 14523	Suppl. material 1: fig. S60	Sabaj Pérez and Birindelli 2008
*Leptodoraspraelongus* (Myers & Weitzman, 1956) ^3^	MPUJ 16518	Suppl. material 1: fig. S61-3C	Sabaj 2005
*Tenellusternetzi* (Eigenmann, 1925) ^3^	MPUJ 15522	Suppl. material 1: fig. S62	Birindelli and de Souza 2018
**Heptapteridae**			
*Leptorhamdianocturna* (Myers, 1928) ^3^	Uncatalogued, photo voucher only	Suppl. material 1: fig. S63	Bockmann and Slobodian 2018
*Mastiglanisasopos* Bockmann, 1994	MPUJ 14392, 14469	Suppl. material 1: fig. S64	Bockmann and Slobodian 2018
*Pimelodella* sp.	MPUJ 14402	Suppl. material 1: fig. S65	Bockmann and Slobodian 2018
**Loricariidae**			
*Ancistruspatronus* de Souza, Taphorn & Armbruster, 2019 ^3^	MPUJ 14470, 14482	Suppl. material 1: fig. S66	de Souza et al. 2019
*Hemiancistrus* sp. ^2^	MPUJ 14509, 14519, 14520	Suppl. material 1: fig. 4F	Werneke et al. 2005
*Loricariacataphracta* Linnaeus, 1758	MPUJ 14401	Suppl. material 1: fig. S68	Isbrücker 1981; Londoño-Burbano et al. 2021
*Rineloricariacachivera* Urbano-Bonilla, Londoño-Burbano & Carvalho, 2023 ^2^	MPUJ 14375, 14451, 14481, 14495	Suppl. material 1: fig. S70	Urbano-Bonilla et al. 2023
*Rineloricaria* sp. 1	MPUJ 14380, 14530	Suppl. material 1: fig. S69	Urbano-Bonilla et al. 2023
**Pimelodidae**			
*Pimelodusalbofasciatus* Mees, 1974	MPUJ 14479, 14503	Suppl. material 1: fig. S71	Rocha and Zuanon 2013
*Pimelodusornatus* Kner, 1858	MPUJ 14518	Suppl. material 1: fig. S72	Rocha and Zuanon 2013
**Pseudopimelodidae**			
*Pseudopimelodusbufonius* (Valenciennes, 1840)	Uncatalogued, photo voucher only	Suppl. material 1: fig. S73	Shibatta and van der Sleen 2018
**Trichomycteridae**			
*Haemomastervenezuelae* Myers, 1927 ^3^	MPUJ 14396, 14465	Suppl. material 1: fig. S74	Fernández 2018
*Ochmacanthusreinhardtii* (Steindachner, 1882)	MPUJ 14387, 14431	Suppl. material 1: fig. S75	Fernández 2018
**GYMNOTIFORMES**			
**Sternopygidae**			
*Archolaemus* sp. ^2^	Uncatalogued, photo voucher only	Suppl. material 1: fig. 4E	Vari et al. 2012
*Eigenmanniamatintapereira* Peixoto, Dutra & Wosiacki, 2015^1^	MPUJ 14420, 14501	Suppl. material 1: fig. S77-3D	Peixoto et al. 2015
*Eigenmannia* sp.	MPUJ 14393	Suppl. material 1: fig. S78	Peixoto et al. 2015
*Sternopygusmacrurus* (Bloch & Schneider, 1801)	Uncatalogued, photo voucher only	Suppl. material 1: fig. S79	Hulen et al. 2005
**GOBIIFORMES**			
**Eleotridae**			
*Microphilypnusternetzi* Myers, 1927 ^2^	MPUJ 14466	Suppl. material 1: fig. S80	Caires and Toledo-Piza 2018
**BLENIIFORMES**			
**Belonidae**			
*Potamorrhaphisguianensis* (Jardine, 1843)	MPUJ 14508	Suppl. material 1: fig. S81	Sant’Anna et al. 2012
**Cichlidae**			
*Aequidensdiadema* (Heckel, 1840) ^2^	MPUJ 14454, 14458, 14459, 14490, 14552	Suppl. material 1: fig. S82	Kullander and Ferreira 1990; Kullander et al. 2018
*Aequidenstetramerus* (Heckel, 1840) ^2^	MPUJ 14459	Suppl. material 1: fig. S83	Kullander and Ferreira 1990; Kullander et al. 2018
*Apistogramma* sp. 1	MPUJ 14450, 14471, 14549, 14372	Suppl. material 1: fig. S84	Kullander et al. 2018
*Apistogramma* sp. 2	MPUJ 14378, 14406, 14551, 14445	Suppl. material 1: fig. S85	Kullander et al. 2018
*Cichlatemensis* Humboldt, 1821	MPUJ 14510	Suppl. material 1: fig. S86	Kullander and Ferreira 2006
*Saxatiliaalta* (Eigenmann 1912) ^2^	MPUJ 14474, 14532	Suppl. material 1: fig. S87	Ploeg, 1991; Varella et al. 2023
*Lugubrialenticulata* (Heckel 1840)	MPUJ 14505	Suppl. material 1: fig. S88	Ploeg, 1991; Kullander and Varella 2015; Varella et al. 2023
*Geophagusabalios* López-Fernández & Taphorn, 2004	MPUJ 14381, 14404, 14415, 14468, 14513, 14526	Suppl. material 1: fig. S89	López-Fernández and Taphorn 2004
**Rivulidae**			
*Anablepsoides* sp.	MPUJ 14485	Suppl. material 1: fig. S90	Amorim and Bragança 2018
**SYNBRANCHIFORMES**			
**Synbranchidae**			
*Synbranchusmarmoratus* Bloch, 1795	MPUJ 14500	Suppl. material 1: fig. S91	Van Der Sleen and Albert 2017
**ACANTHURIFORMES**			
**Sciaenidae**			
*Pachyurusgabrielensis* Casatti, 2001	MPUJ 14412, 14441	Suppl. material 1: fig. S92	Casatti 2001
*Pachyurusjunki* Soares & Casatti, 2000	MPUJ 14511	Suppl. material 1: fig. S93	Casatti 2001
*Pachyurusschomburgki* Gunther, 1860	MPUJ 14512	Suppl. material 1: fig. S94	Casatti 2001
*Plagioscionsquamosissimus* (Haeckel, 1840)	Uncatalogued, photo voucher only	Suppl. material 1: fig. S95	Casatti 2005

### ﻿First records of species photographs in life

This article is one of the first to implement a workflow that associates photographs of live specimens in the field with the meticulous taxonomy carried out in the laboratory and its subsequent upload to the CaVFish Project, Colombia. Many of these species did not have adequate visual records in life, and therefore this study represent a great advance in the knowledge of the ichthyofauna of the Vaupés River, both for specialists and for the broader public. All species photographed can be accessed from the project page using the following URL: https://cavfish.unibague.edu.co.

### ﻿New records for Colombia

**Figure 3.**
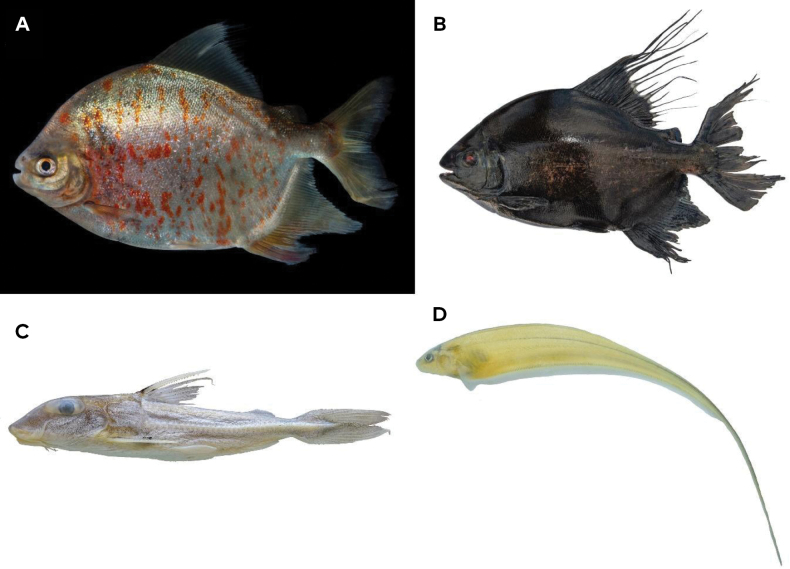
New records for Colombia **A***Mylopluslucienae* 335.1 mm SL **B***Tometesmakue* 395 mm SL lost and uncatalogued **C***Leptodoraspraelongus* 175.2 mm SL **D***Eigenmanniamatintapereira* 249.1 mm SL.

This study records for the first time in Colombia the following four species: *Mylopluslucienae* Andrade, Ota, Bastos & Jégu, 2016, *Tometesmakue* Jégu, Santos & Jégu, 2002 also a first record of the genus, *Leptodoraspraelongus* (Myers & Weitzman, 1956), and *Eigenmanniamatintapereira* Peixoto, Dutra & Wosiacki, 2015. These species were absent from recent lists of fish species of Colombia (DoNascimiento et al. 2017, 2024; Bogotá Gregory et al. 2020, 2022a) (Fig. 3A–D).

#### ﻿*Mylopluslucienae* Andrade, Ota, Bastos & Jégu, 2016

Specimens collected in this expedition contributed three lots (MPUJ 14524-3 spec.; 14525-1 spec.; 14528-1 spec.; Fig. 3A; Table 3). This species was recently described, and its known distribution was restricted to the Negro River between Manaus and São Gabriel da Cachoeira in Brazil (Andrade et al. 2016). This species is found in blackwater rivers and typically inhabits rapids. *Mylopluslucienae* can be distinguished from other congeners by the combination of an elongated body, small pre-pelvic spines that reach anteriorly just to the middle of the abdomen, and large scales on flanks resulting in lower scale counts (Andrade et al. 2016).

#### ﻿*Tometesmakue* Jégu, Santos & Jégu, 2002

There are five lots from our expedition (MPUJ 14498-7 spec.; 14527-1 spec.; 14529-1 spec.; 14550-1 spec.; 14553-1 spec.; Fig. 3B; Table 3). This species is known to occur in the Negro and Orinoco River basins. In the Negro River, it was reported in several tributaries, including the Rio Uaupés in Brazil at Cachoeira de Ipanoré (Jégu et al. 2002). These represent the first records upstream of that location within Colombian territory. This species is diagnosed among its congeners by the combination of great number of teeth in the inferior jaw (11 teeth) in specimens greater than 100 mm SL in comparison to congeneric species; fewer pre-pelvic serrae (1–9), total serrae (10–23), and horizontal mouth.

#### ﻿*Leptodoraspraelongus* (Myers & Weitzman, 1956)

There is a single lot from our expedition (MPUJ 16518-2 spec.; Fig. 3C; Table 3). This species is known from blackwater drainages in Brazil and Venezuela, associated with large river cataracts on the upper Orinoco and Negro rivers, and occurs in several localities along the Amazon River (Sabaj 2005). This species is diagnosed based on the combination of the following characters: half of dorsal fin without a black spot or blotch; dorsal spine not extended as a long flexible filament; absence of dark nuchal saddle; and flap-like posterior extensions at corners of lower lip narrow and long, finishing beyond tips of similar extensions at corners of upper lip; sum of midlateral plates usually < 75 (range 70–76) and anterior midlateral plates shallow; height of 2^nd^ midlateral plate less than or equal to vertical diameter of eye; anterior nuchal plate usually narrow, permitting suture between supraoccipital and middle nuchal plate; profile of snout weakly concave (Sabaj 2005).

#### ﻿*Eigenmanniamatintapereira* Peixoto, Dutra & Wosiacki, 2015

There are two lots from our expedition (MPUJ 14420-1 spec.; 14501-4 spec.; Fig. 3D; Table 3). The species was described from the Negro River in Brazil (Peixoto et al. 2015) and previous records were known from the Uneiuxi and Urubaxi rivers, tributaries to the Negro River, near Santa Isabel do Rio Negro in Brazil. This species is diagnosed among species of the *Eigenmanniatrilineata* group López & Castello, 1966 by having the pectoral fin with a dusky coloration or with a conspicuous dark blotch, and 16-17 branched pectoral-fin rays (Peixoto et al. 2015).

### ﻿Presumably undescribed species

**Figure 4.**
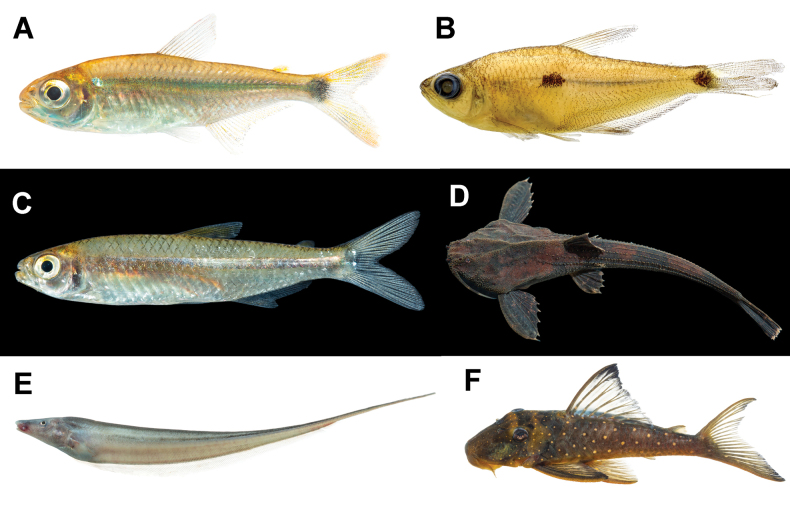
Presumably undescribed species **A***Jupiaba* sp. 32.6 mm SL **B***Phenacogaster* sp. 31.3 mm SL **C***Moenkhausia* sp. 33.1 mm SL **D***Bunocephalus* sp. 42.5 mm SL **E***Archolaemus* sp. Lost and uncatalogued copy **F***Hemiancistrus* sp. 115.3 mm SL.

We found six undescribed species in the lower Vaupés River in Colombia. When verifying their diagnostic characters, they did not coincide with recent taxonomic revisions of each of the genera (Fig. 4A–F). There are genera such as *Eigenmannia* Jordan & Evermann, 1896, *Knodus* Eigenmann, 1911, and *Apistogramma* Regan, 1913 that require taxonomic revisions and under further scrutiny may represent undescribed species.

#### ﻿*Jupiaba* Zanata, 1997

There are several lots from our expedition of an undescribed species of *Jupiaba* (MPUJ 14385-13 spec.; 14424-3 spec.; 14440-1 spec.; 14446-8 spec.; 14467-5.; 14475-8 spec.; 14488-2 spec.; 14538-1 spec.; 14370; Table 3). This species of *Jupiaba* (Fig. 4A) is most similar to *J.atypindi* Zanata, 1997 and *J.poekotero* Zanata & Lima, 2005 by sharing the combination of premaxillary teeth cusps similar in shape and size; dentary teeth gradually decreasing in size posteriorly; third infraorbital not contacting preopercle ventrally; dark humeral blotch vertically elongated, bordered by clear areas; and teeth of the inner series of premaxilla usually with 7, 9, or 11 cusps (Netto-Ferreira et al. 2009). It differs from *J.atypindi* and *J.poekotero* by its shallower body and distinct coloration pattern on the caudal fin (caudal blotch not reaching ventral and lower margin of caudal peduncle and caudal rays mostly hyaline).

#### ﻿*Phenacogaster* Eigenmann, 1907

There are two lots from our expedition (MPUJ 14390-5 spec.; MPUJ 14364-1 spec.; Table 3) of an undescribed species of *Phenacogaster* (Fig. 4B). This species has a unique posteriorly displaced humeral spot at a level below dorsal-fin origin that is similar to *P.tegatus* (Eigenmann, 1911), a species distributed in the Paraguay River basin (Lucena and Malabarba 2010). Differing from *P.tegatus*, this species has a complete lateral line (vs an incomplete lateral line).

#### ﻿*Moenkhausia* Eigenmann 1903

There are seven lots from our expedition (MPUJ 14542-11 spec.; 14543-2 spec.; 14408-13 spec.; 14411-5 spec.; 14427-7 spec.; 14443-7 spec.; 14374-3 spec.; Table 3) of an undescribed species belonging to the *Moenkhausialepidura* group (Kner, 1858; Fig. 4C). Specimens of this species are similar to *Moenkhausiahasemani* Eigenmann, 1917 also in the *M.lepidura* group by having the combination of predorsal scales arranged in a single median row; it has a humeral spot, conspicuous, which is narrow, vertically elongated, and located on the third to fifth lateral-line scale; five longitudinal scale rows above the lateral line and with 34 or 35 perforated scales on the lateral line; unbranched dorsal-fin rays hyaline, and a longitudinal dark line extending from the humeral spot (or slightly posterior to it), becoming wider at vertical through the posterior third of dorsal fin to the caudal peduncle; caudal-fin lobe mark variable, frequently presenting a semicircular darker spot on its middle portions, and faintly on middle caudal-fin rays (Marinho and Langeani 2016). It differs from *M.hasemani* by having a lower anal-fin ray count with 17–20 branched rays (mode 19) contrasting with 20–23 branched rays in *M.hasemani* (M. Marinho pers. comm. 29 Nov 2021).

#### ﻿*Bunocephalus* Kner, 1985

There is a single record from our expedition (MPUJ 14433-1 spec; Table 3) of an undescribed species of *Bunocephalus* (Fig. 4D). Species delimitation in *Bunocephalus* is based mainly on morphometric characters and a few meristic features such as fin-ray counts (Mees 1989; Carvalho et al. 2015). The collected species in the Vaupés River represents a species previously identified in a phylogeny whose geographic distribution is the upper Negro and Orinoco river basins (Carvalho et al. 2018); the species could not be identified to species level based on the current literature and likely represents an undescribed species.

#### ﻿*Archolaemus* Korringa, 1970

The specimen illustrated in Fig. 4E is the first record of the genus *Archolaemus* in Colombia. Unfortunately, it was lost and not catalogued, and there is only a photographic record and a tissue sample (MPUJ_P_T3796) representing this specimen. The genus *Archolaemus* was reviewed by Vari et al. (2012), and its six known species are distributed in Amazon tributaries draining the Brazilian and the Guiana Shields and in the São Francisco River basin in Brazil. Each species of *Archolaemus* is endemic to a single basin and the geographically closest records of *Archolaemus* to the Vaupés River are of *A.ferrerai* Vari, de Santana & Wosiacki, 2012 in the Uraricoera, a tributary of the Branco River, Negro River basin, Brazil. Based on the photo voucher, the species is most similar to *A.luciae* Vari, de Santana & Wosiacki, 2012 in sharing the combination of traits with a large mouth extending posterior to a vertical through the posterior naris, and a caudal-appendage depth of 3.3–4.8% of the caudal appendage length, which is ~ 4.7% SL. It can be tentatively distinguished from *A.luciae* by having more anal-fin rays (216 vs 192–213; Vari et al. 2012). Given its disjunct geographic distribution from *A.luciae* and other species of the genus, this new record may represent an undescribed species.

#### ﻿*Hemiancistrus* Bleeker, 1862

There are several records from our expedition (MPUJ 14509-4 spec.; 14519-4 spec.; 14520-1 spec.; Table 3) of an undescribed species of *Hemiancistrus* (Fig. 4F). This species looks like *H.subviridis* Werneke, Sabaj Pérez, Lujan & Armbruster, 2005 by the shared presence of golden yellow spots on the body, but contrasts with *H.subviridis*; however, the species has spots distributed all over the body (vs spots concentrated in the anterior half of the body) and a conspicuous darker posterior margin of the dorsal fin.

## ﻿Discussion

The Amazon Basin has the greatest freshwater fish biodiversity on the planet (Tisseuil et al. 2013; Dagosta and de Pinna 2019). The Negro River basin and its main drainages have been explored for the last three centuries (Lima et al. 2005). Historical analyses (1821–2019), however, suggest that species richness in the Negro basin is far from being fully known, given that the rate of species descriptions has not stabilized (Beltrão et al. 2019) and there are still areas unexplored scientifically (Jézéquel et al. 2020a). In the Brazilian part of the basin, the uniqueness of the headwater ichthyofaunas are well-documented (Lima et al. 2005), resulting in recent descriptions of more than 30 new species (Beltrão et al. 2019; Bogotá-Gregory et al. 2022a). Recent rigorous work resulted in recording 1,165 fish species associated with different aquatic environments in the basin (Beltrão et al. 2019). Of this compilation, Bogotá-Gregory et al. (2022a) recorded 224 species in the middle Vaupés River basin, of which ten are new records for Colombia and 26 are new records for the Colombian Amazon basin. Our research in the lower Vaupés River basin adds four new records for Colombia and 44 new records (see Table 3) not included in Bogotá-Gregory et al. (2022a), resulting in 268 fish species now known in the middle and lower portions of the Vaupés River basin.

Based on these recent lists of fish species composition (Beltrão et al. 2019; Bogotá-Gregory et al. 2022a) and our results, the entire Negro River basin reaches an approximate richness of 1,210 species. This richness value is still under the predicted estimates that vary between 1,466 and 1,759 species (Beltrão et al. 2019). Despite this, our expedition revealed new records for Colombia, and undescribed species to science, demonstrating that fish diversity in the region is still far from completely known (Bogotá-Gregory et al. 2020).

Although the Vaupés drainages located to the southwest of our study area have been well sampled (rivers Papuri, Cuduyari Paca, Mituceño, and Tiquié), this study adds new records for the country. Therefore, it is essential to continue monitoring fishes from rheophilic environments and especially those that live in the headwaters of the Vaupés (e.g., Itilla and Unilla rivers; see Fig. 1). This area is recognized for its high degree of species endemism (Hernández-Camacho et al. 1992), the singularity of its fish fauna (Londoño-Burbano and Urbano-Bonilla 2018; Lima et al. 2020), and its connectivity with two protected natural area, the Serranías de la Macarena and Chiribiquete National Parks (Botero and Serrano 2019).

Two new records for Colombia are represented by the serrasalmids *Mylopluslucienae* and *Tometesmakue*. From the expeditions of Alfred Russel Wallace (1850–1852) along the Vaupés River, there are illustrations of 43 specimens, representing ~ 40 serrasalmid species (Toledo-Piza 2002). Of these, the fish named “pacu-muritinga” and “pacu-tinga” came to be recognized as *Mylopluslucienae*, a species associated with both rapids and more slowly running waters (Andrade et al. 2016). Therefore, despite the long-known occurrence of this species downstream in the Negro River, this is the first record of this species upstream in the Colombian portion of this basin.

Before the present record of *Mylopluslucienae* in Colombia, the genus was represented by four species in the country, *M.asterias* (Müller & Troschel, 1844), *M.rubripinnis* (Müller & Troschel, 1844), *M.schomburgkii* (Jardine, 1841), and *M.torquatus* (Kner, 1858). The genus *Tometes*, however, was not yet recorded in Colombia (Bogotá-Gregory et al. 2022a; DoNascimiento et al. 2024). The populations of *M.lucienae* are distributed in the Negro River basin in Brazil (Andrade et al. 2016) and those of *Tometesmakue* in the middle and upper basin of the Negro River in Brazil and the Orinoco River in Venezuela (Jégu et al. 2002); we now document for Colombia the sympatric occurrence of *M.lucienae* (Fig. 3A) and *T.makue* (Fig. 3B). These fishes live in rocky rapids preferably associated with habitats with abundant aquatic vegetation (Podostemaceae). Sympatric fish assemblages form through dispersal and ecological coexistence (Thomaz et al. 2019; Albert et al. 2020).

In *T.makue*, the stomach contents of adult specimens reveal that Podostemaceae plants represent a very important part of the diet of these fishes (Jégu et al. 2002; Lima et al. 2005). On the other hand, *Mylopus* species are generalist herbivorous, with seeds as the main food source, and occasionally feeding on small aquatic animals (van der Sleen and Albert 2017; Correa and Winemiller 2018). In an analysis of the evolution of the diet in the Serrasalmidae family, associated changes in dentition highlight ecomorphological diversity (Kolmann et al. 2021). Podostemaceae makes up most of the diet (based on relative volume) of *Tometes* compared with *Mylopus* (Kolmann et al. 2021), which may explain their sympatric existence.

Between 1923 and 1925 ichthyologist Dr. Carl Ternetz traveled the Amazon from Manaus, up the Negro River and across to the Orinoco River, accruing collections that resulted in descriptions of several new species (Lima et al. 2005). During this expedition, the collected specimens of a fish would be described as *Hassarpraelongus* Myers & Weitzman, 1956 (currently *Leptodoraspraelongus*) 38 years later, distributed in Brazil and Venezuela (Sabaj 2005; Beltrão et al. 2019). Species of the genus *Leptodoras* are widely distributed in the Amazon, Orinoco and Essequibo River basins (Sabaj 2005; Birindelli et al. 2008; van der Sleen and Albert 2017; Taphorn et al. 2022). In the Negro River basin (Brazil), seven species [(*Leptodorasacipenserinus* (Günther, 1868), *L.cataniai* Sabaj Pérez, 2005, *L.copei* (Fernández-Yépez, 1968), *L.hasemani* (Steindachner, 1915), *L.juruensis* Boulenger, 1898, *L.linnelli* Eigenmann, 1912, and *L.praelongus* (Myers & Weitzman, 1956)] are known (Beltrão et al. 2019) while for the entire Colombian Amazon five species are known [(*L.acipenserinus*, *L.copei*, *L.juruensis*, *L.linnelli*, and *L.myersi* Böhlke, 1970; Bogotá-Gregory et al. 2022a; DoNascimiento et al. 2024)]. Of these, four are shared with drainages of the Colombian Amazon and the Negro River (*L.acipenserinus*, *L.copei*, *L.juruensis*, and *L.linnelli*); two species live in this last river (*L.cataniai* and *L.hasemani*) that are absent in the Colombian Amazon, and that does have records of *L.myersi*, currently absent in the Negro River (Beltrão et al. 2019; DoNascimiento et al. 2024). In the rapids of the Macucú community (Mitú), a single specimen of *Leptodoraspraelongus* (Fig. 3C) was collected from benthic habitats in deep, fast flowing waters. Some species in the genus (e.g., *L.juruensis* and *L.myersi*) are restricted to deep habitats (50 m; Sabaj 2005). *Leptodoraspraelongus* possibly lives in sympatry with *L.copei*, recorded for the middle Vaupés River basin (Bogotá-Gregory et al. 2022a), contrasting with L.cf.linnelli that lives downstream in the rapids of Carurú, at the border between Brazil and Colombia (Lima et al. 2005).

Within the electric glassfishes, we recorded new species for Colombia in the genus *Eigenmannia* Jordan & Evermann, 1896. This genus represents the most diverse group in the family Sternopygidae and is distributed throughout Central and South America (Fricke et al. 2023), with its greatest diversity in the Amazon basin (Peixoto and Ohara 2019). It has 24 valid species distributed into two groups; one is called *E.trilineata* group, which includes 22 species (Dutra et al. 2022), with a complex taxonomy, and until recently, *E.virescens* (Valenciennes, 1836) and *E.trilineata* López & Castello, 1966 were erroneously cited as occurring in several Amazon basin drainages. We indicate the presence of two species belonging to the *E.trilineata* group: *E.matintapereira* and an unidentified species. Although there are specimens identified as *Eigenmannia* sp. in other recent inventories in the region (Lima et al. 2005: 256; Bogotá-Gregory et al. 2022a), it is difficult to confirm if these species belong to *E.matintapereira* or even the *E.trilineata* group. Despite that, the present study highlights the sympatry of at least two morphotypes of *Eigenmannia* that occur in the lower basin of the Vaupés River associated with rocky rapids and sandy beaches.

Oberdorff et al. (2019) evaluated 97 Amazon basin drainages and found the size of the habitat, the modern and past climates, and isolation due to natural waterfalls contribute to explain patterns of endemic richness. Naturally, the Vaupés River and the breaks in the relief represented by numerous rapids are common in some of its main drainages. An example of this is the Tiquié River, a tributary of the Vaupés (Fig. 1), which in its route through the different rapids (i.e., Pari-Cachoeira, Pedra Curta, Comprida, and Carurú) shows gradients in fish communities in the downstream-upstream direction; upstream of the Carurú rapids, the absence of some genera (*Phenacogaster* Eigenmann, 1907 and *Serrasalmus* Lacepède, 1803) or species [(*Moenkhausiacollettii* (Steindachner, 1882), *Anduzedorasoxyrhynchus* (Valenciennes, 1821), *Pseudoplatystomatigrinum* (Valenciennes, 1840), *Ageneiosusinermis* (Linnaeus, 1766)] (Lima et al. 2005) is evident. This seems to be consistent with our results, with the exception of *A.inermis*, which is one of the 92 species of fish identified by the inhabitants of the Tiquié communities in Colombia (Campuzano-Zuluaga 2019).

Contrary to what was observed in the upper part of the Tiquié River, the rapids of the Vaupés River in Colombia (e.g., Fig. 1: rapids upstream between the Colombia-Brazil border to the town of Mitú: Carurú, Matapí, Tapira Geral, La Mojarra, Macucú, Nana, Villa Fátima rapids) present a different pattern in the occurrence of species since most species listed above are also found in this part of the drainage. In this sense, the rapids at the headwaters of the Vaupes River possibly act as dispersal filters for some species of fishes.

Records are located in the Vaupés Arc, a Miocene origin arch that represents the divide between the Amazon-Orinoco river basins, and represents a semi-permeable barrier for fish dispersal (Winemiler and Willis 2011: table 14.3). Anecdotally, local communities refer to an absence of species upstream of the rapids of Carurú (1°5'8.81"N, 69°19'39"W) that constitutes an important barrier for fish dispersal. An example is the absence of freshwater stingrays *Potamotrygon* Garman, 1877), the electric eel (*Electrophorus* Gill, 1864), and large migratory catfish (*Brachyplatystoma* Bleeker, 1862) as evidenced in Table 3 and previously published lists of the middle Vaupés River basin in Colombia (Bogotá‐Gregory et al. 2022a). In our expedition, we sampled only upstream of this barrier and we did not collect any of these groups.

From another territorial perspective, the historical and traditional knowledge of indigenous communities makes it possible to identify the anthropic displacement of species for subsistence purposes in the Amazon basin (Lima et al. 2005; Camacho-García 2013; Campuzano-Zuluaga 2019). In 1950, in the upper Tiquié River basin, the community transported upstream of the Pedra Curta rapids a fish for consumption, *Satanopercajurupari* (Heckel, 1840), and it was anticipated that it would colonize the headwaters of the river on the Colombian side (Lima et al. 2005). The coexistence of this species with locally native fish was confirmed 14 years later (Campuzano-Zuluaga, 2019). In addition, it was known that this species was already found naturally in the middle basin of the Vaupés (Bogotá-Gregory et al. 2022a) including its headwaters, i.e., the Unilla and Itilla rivers (Prada-Pedreros et al. 2018). Likewise, in 1990 another fish used by the communities, *Lugubriajohanna* (Heckel, 1840) was transported from the Japurá-Caquetá River basin (in Brazil) to the headwaters of the Tiquié (on the Colombian side) (Lima et al. 2005; Campuzano-Zuluaga 2019) and today inhabits the entire Vaupés River basin, including the main channel, lagoons and main drainages (Beltrão et al. 2019).

Transporting fish species among subbasins of the upper Vaupés River in Colombia threatens both the aquatic biodiversity and the fisheries production of this region. The historical and traditional records reveal the introduction of non-native species, mostly cichlids [(e.g., *Lugubriajohanna*, *Heros* sp., *Mesonautainsignis* (Heckel, 1840), *Satanopercajurupari*)] and an Erythrinidae (*Hopliaslacerdae* Ribeiro, 1908) in the upper Tiquié (Lima et al. 2005; Campuzano-Zuluaga, 2019) that could be related to the decline in the populations of the region’s native fish fauna, and threaten the security and food security of the peoples present there (Campuzano-Zuluaga, 2019). Although these species are widely distributed in the Amazon, Orinoco, and Guyanese Shield basins (Beltrão et al. 2019), historical data show translocation of these fish in areas where they did not occur before, and isolated to a certain extent by a series of rapids but living in sympatry with natural populations (Lima et al. 2005).

Regarding cichlids, an example of the extinction of the endemic fauna is known when *Latesniloticus* (Linnaeus, 1758) was introduced to Lake Victoria (Witte et al. 1992). Species checklists and photos document the composition of fish (native and non-native) of the upper Tiquié River, which rises in the southeast of the Colombian territory, in a lagoon system called Ewura (Campuzano-Zuluaga 2019), on its way through Brazilian territory they give way to countless rapids that can act as barriers (Lima et al. 2005). In recent years, endemic (native) fish species have recently been discovered from specific areas of the Tiquié River basin in Brazil [(e.g., *Corydorasdesana* Lima & Sazima, 2017; *Hypostomuskopeyaka* and *H.weberi* Carvalho, Lima & Zawadzki, 2010, *Rhinotocinclusyaka* (Lehmann A., Lima & Reis, 2018)] and from the Vaupés River in Colombia (i.e., *Rineloricariajurupari* Londoño-Burbano & Urbano-Bonilla, 2018, *Hemigrammusxaveriellus* Lima, Urbano-Bonilla, Prada-Pedreros, 2020 and *Rineloricariacachivera* Urbano-Bonilla, Londoño-Burbano & Carvalho, 2023). The introduction of non-native fish such as Cichlids generates irreversible effects (displacement, extinction of species, and loss of the gene pool of native species) due to (intraspecific) competition and direct predation (Ogutu‐Ohwayo 1993).

In the Amazon Basin, sub-basins with greater accessibility (i.e., shorter travel times from cities or closer to river ports) generally experience greater inventory effort in terms of location density and number of years inventoried, if compared to sites with less accessibility, which is one of the main limitations in fish inventories (Herrera-R et al. 2023). In the study of fish from different geomorphic habitats of the Amazonian lowlands (rivers, alluvial plains, terra firme streams, and shield streams), it is suggested to consider the temporal dynamics of habitat types and variation in hydrological seasonality (Bogotá-Gregory et al. 2023). In this sense, the basin of the Vaupés River in Colombia offers unique and incomparable study opportunities due to its remote and difficult-to-access location, in addition to its geological history, temporal and spatial variability created by rapids, make these results fill gaps of information in areas never before sampled.

The Vaupés River born in the foothills of the eastern Colombian mountain range and flows through outcrops of the Guiana Shield and sandy-soils of the Amazonian lowlands. The water chemistry of this basin is therefore a combination of sediment-rich Andean whitewaters (Unilla river sub-basin; see Fig. 1) and acidic blackwaters that drain sandy lowland rainforest soils (i.e., oxisols) of the peri-Guiana shield region (Itilla River sub-basin; see Fig. 1). The large information gaps in the area (Jézéquel et al. 2020a), the presence of endemic rheophilic species (*Rineloricariajurupari* Londoño-Burbano & Urbano-Bonilla, 2018, *R.daraha* Rapp Py-Daniel & Fichberg, 2008, *R.cachivera* Urbano-Bonilla Londoño-Burbano & Carvalho, 2023), and various undescribed species (*Odontocharacidium* Buckup, 1993, *Tetragonopterus* Cuvier, 1816, *Tyttocharax* Fowler, 1913, *Ituglanis* Costa & Bockmann, 1993, *Myoglanis* Eigenmann, 1912, *Nemuroglanis* Eigenmann & Eigenmann, 1889 and *Aequidens* Eigenmann & Bray, 1894; Bogotá-Gregory et al. 2022a) including those in this study (i.e., *Jupiaba* sp., *Moenkhausia* sp., *Phenacogaster* sp., *Bunocephalus* sp., *Hemiancistrus* sp., and *Archolaemus* sp.) support the need to strengthen scientific expeditions and community monitoring of fish. This research should be accomplished in partnership with local indigenous communities or settlers that depend on fish for their subsistence, especially those who live in the rapids of the Vaupés River (i.e., Carurú, Matapí, Tapira Geral, La Mojarra, Macucú, Nana, Villa Fátima, and its headwaters, the Itilla and Unilla).

## ﻿Conclusions

This study contributes new fish records for the Vaupes Arch region, a biodiverse but poorly explored region of the Colombian Amazon of high geological importance with extensive and well-preserved forested and aquatic habitats. Thes results increase the documented fish diversity of this region to 95 species, identify several putatively new species to science, and further document geographic and habitat distribution patterns. Continued systematic sampling of this region at larger spatial and temporal scales will advance progress in the knowledge of the species, populations, communities, and their habitats, especially the rapids of the Vaupés River. The taxonomic lists and high-resolution photographs made available from on public consultation platforms (CaVFfish Project - Colombia), represent important resources for monitoring, conservation, and fisheries management of the Vaupés River basin, at local, regional, national and international levels for waters shared among Brazil, Venezuela and Colombia.

## ﻿Additional information

### Author ORCIDs

Alexander Urbano-Bonilla https://orcid.org/0000-0002-0190-8913

Jorge E. Garcia-Melo https://orcid.org/0000-0003-2885-5652

Mateo Esteban Peña-Bermudez https://orcid.org/0000-0003-3900-3826

Sandra Bibiana Correa https://orcid.org/0000-0003-4466-6923

Tiago P. Carvalho https://orcid.org/0000-0001-5901-1634

### Data availability

All of the data that support the findings of this study are available in the main text or Supplementary Information.

## Supplementary materials

Supplementary material 1 Photographic atlas of voucher specimens Data type docx Explanation note Photographic atlas of voucher specimens collected on the lower Vaupés River, Vaupés, Colombia. Measurements are presented as standard length (SL). All species photographed in life are uploaded to the CaVFish Colombia Project. https://binary.pensoft.net/file/1059082This dataset is made available under the Open Database License (http://opendatacommons.org/licenses/odbl/1.0/). The Open Database License (ODbL) is a license agreement intended to allow users to freely share, modify, and use this Dataset while maintaining this same freedom for others, provided that the original source and author(s) are credited.Alexander Urbano-Bonilla, Jorge E. Garcia-Melo, Mateo Esteban Peña-Bermudez, Omar Eduardo Melo-Ortiz, Oscar Stiven Ordoñez, Sandra Bibiana Correa, Tiago P. Carvalho, Javier A. Maldonado-Ocampo
