# Supplementary material for: ﻿Fishes (Actinopterygii) of the rapids and associated environments in the lower Vaupés River Basin: an undiscovered Colombian Amazon diversity
Source: Zookeys. 2024 May 30;1203:131–58. doi: 10.3897/zookeys.1203.100642 (PMC11161689; doi:10.3897/zookeys.1203.100642)
Supplement: Supplementary material 1 — Photographic atlas of voucher specimens [file zookeys-1203-131_article-100642__-s001.docx]

**Appendix 1.** Photographic atlas of voucher specimens collected on the lower Vaupés River, Vaupés, Colombia. Measurements are presented as standard length (SL). All species photographed in life are uploaded to the [CaVFish Colombia Project](https://experience.arcgis.com/experience/c2709552ee6244a69d96b7fa0d1fdc51/page/Cat%C3%A1logo-de-peces/?views=Vista-10)

  
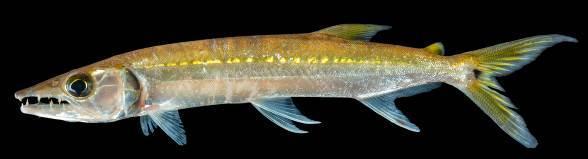


**Figure S1.** *Acestrorhynchus microlepis*, live specimen, MPUJ uncatalogued, 180 mm SL, Vaupés River at Resguardo Trubón.


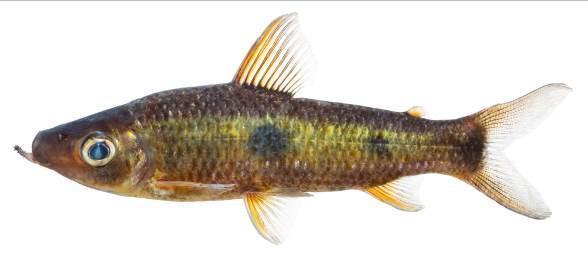


**Figure S2.** *Gnathodolus bidens*, live specimen, MPUJ 14496, 110 mm SL, Vaupés River at Resguardo de Matapí.


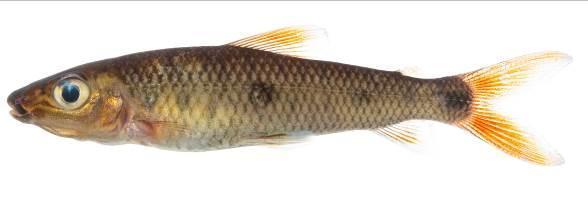
**Figure S3**. *Leporinus brunneus*, live specimen, MPUJ 14504, 155 mm SL, Vaupés River at Resguardo de Matapí.

**
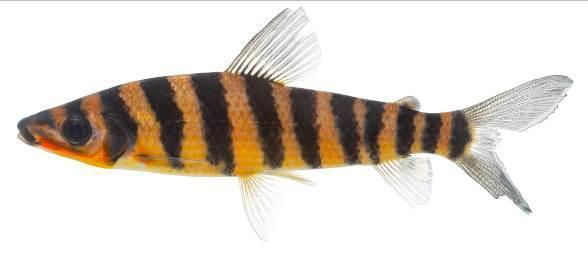
**

**Figure S4.** *Leporinus fasciatus*, live specimen, MPUJ 14478, 123 mm SL, Vaupés River at Resguardo de Matapí.


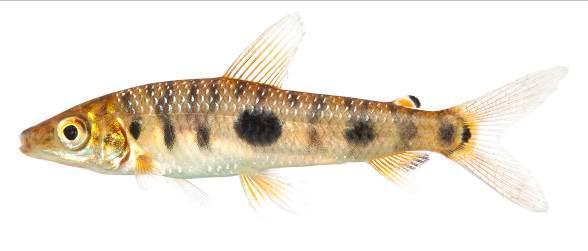


**Figure S5.** *Leporinus niceforoi*, live specimen, MPUJ 14539, 95 mm SL, Vaupés River at Resguardo de Villa Fátima.


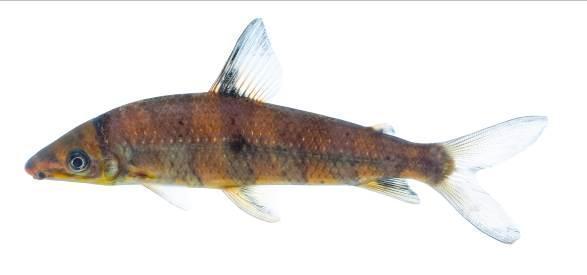


**Figure S6.** *Leporinus yophorus*, live specimen, MPUJ 14506, 140 mm SL, Vaupés Riverat Resguardo de Matapí.


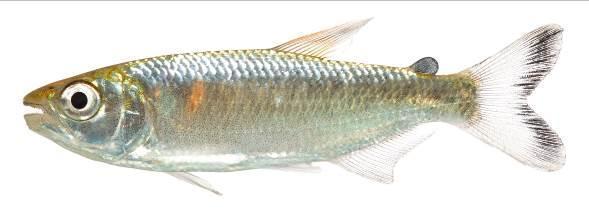


**Figure S7.** *Brycon pesu*, live specimen, MPUJ 14383, 105 mm SL, Vaupés River at Resguardo Trubón.


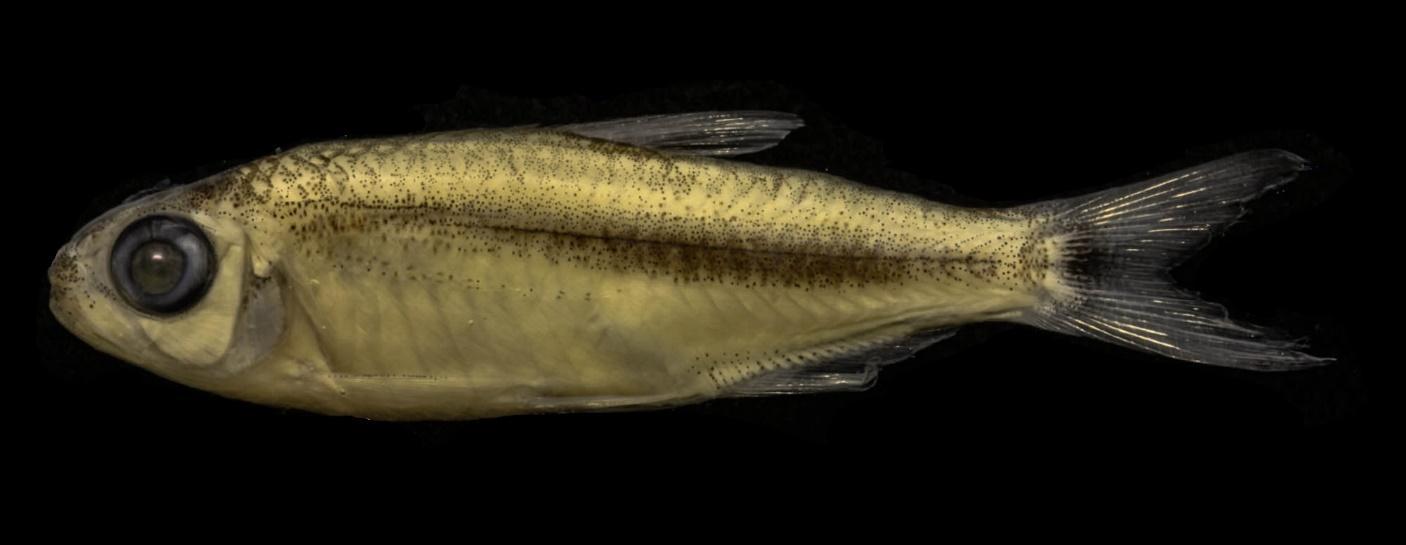


**Figure S8.** *Bryconamericus orinocoensis*, preserved specimen, MPUJ 14386, 58 mm SL, Vaupés River at Villa Fátima.


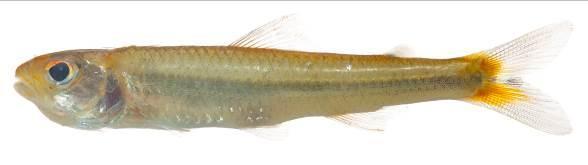


**Figure S9**. *Creagrutus maxillaris*, preserved specimen, MPUJ 14388, 53 mm SL, Vaupés River at Resguardo de Villa Fátima.


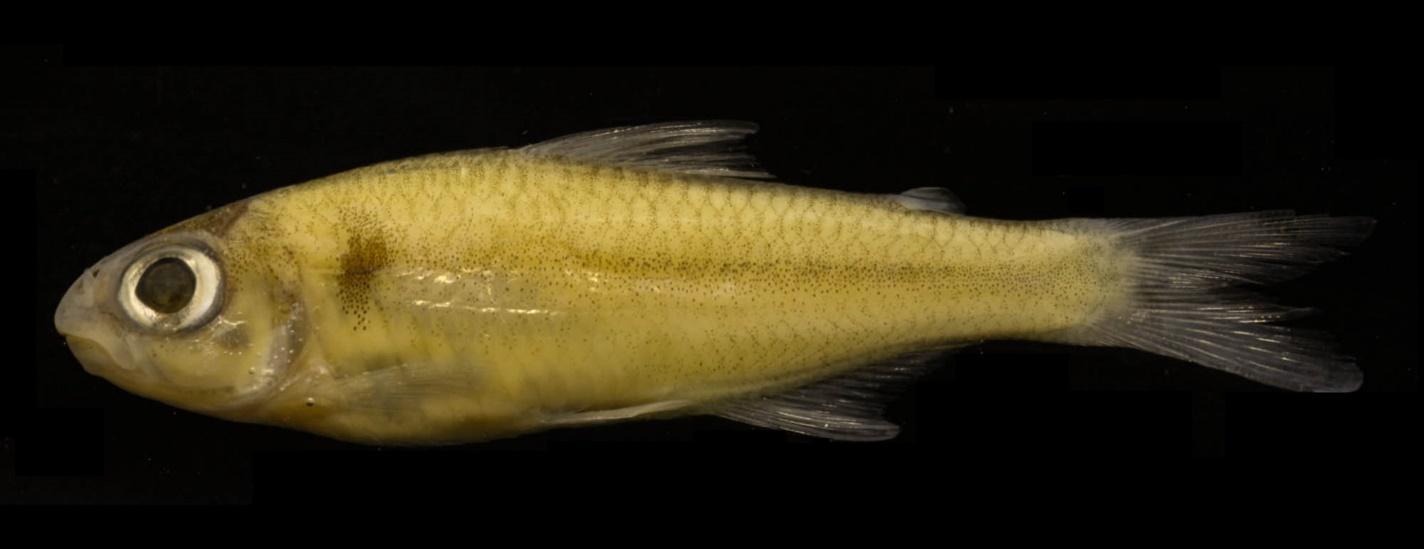


**Figure S10.** *Creagrutus vexillapinnus*, preserved specimen, MPUJ 14394**,** 38 mm SL, Vaupés River at Resguardo de Villa Fátima.

 
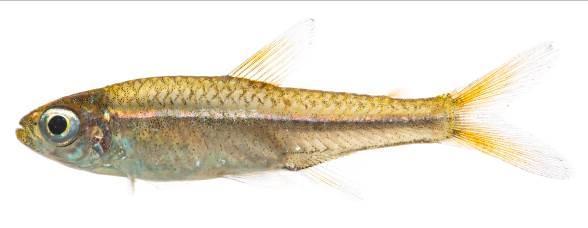


**Figure S11**. *Hemigrammus analis*, live specimen, MPUJ 14486, 42 mm SL, Vaupés River at Resguardo de Matapí.

 
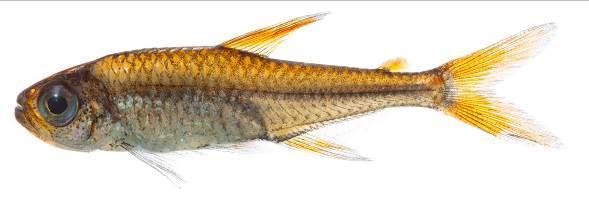


**Figure S12**. *Hemigrammus bellotti*, live specimen, MPUJ 14546, 45.2 mm SL, Caño Danta tributary to Vaupés River at Resguardo de Villa Fátima.


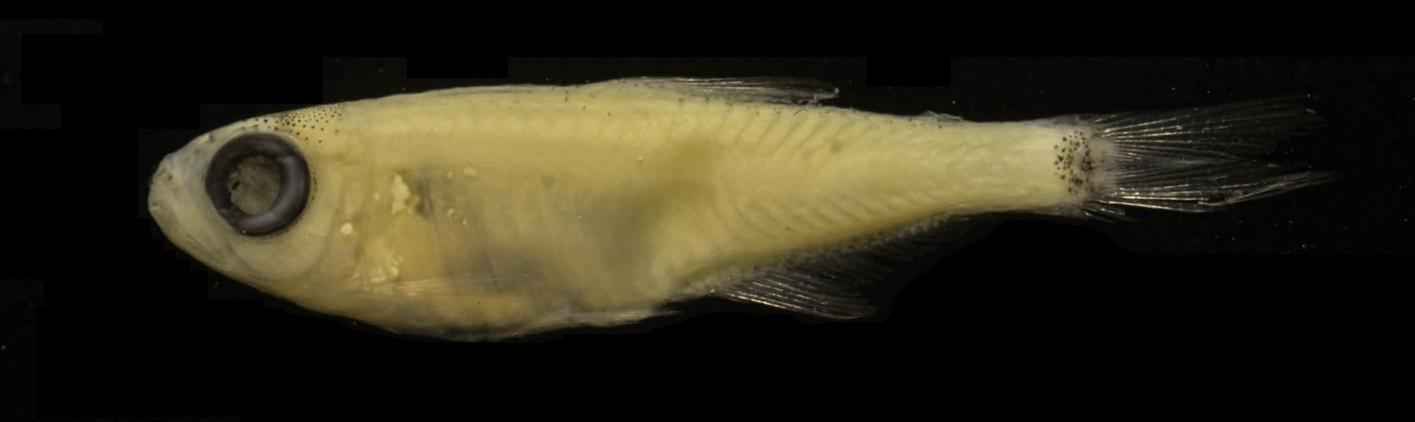


**Figure S13.** *Hemigrammus geisleri*, preserved specimen, MPUJ 14421, 24 mm SL, Vaupés River at Resguardo de Macucú.


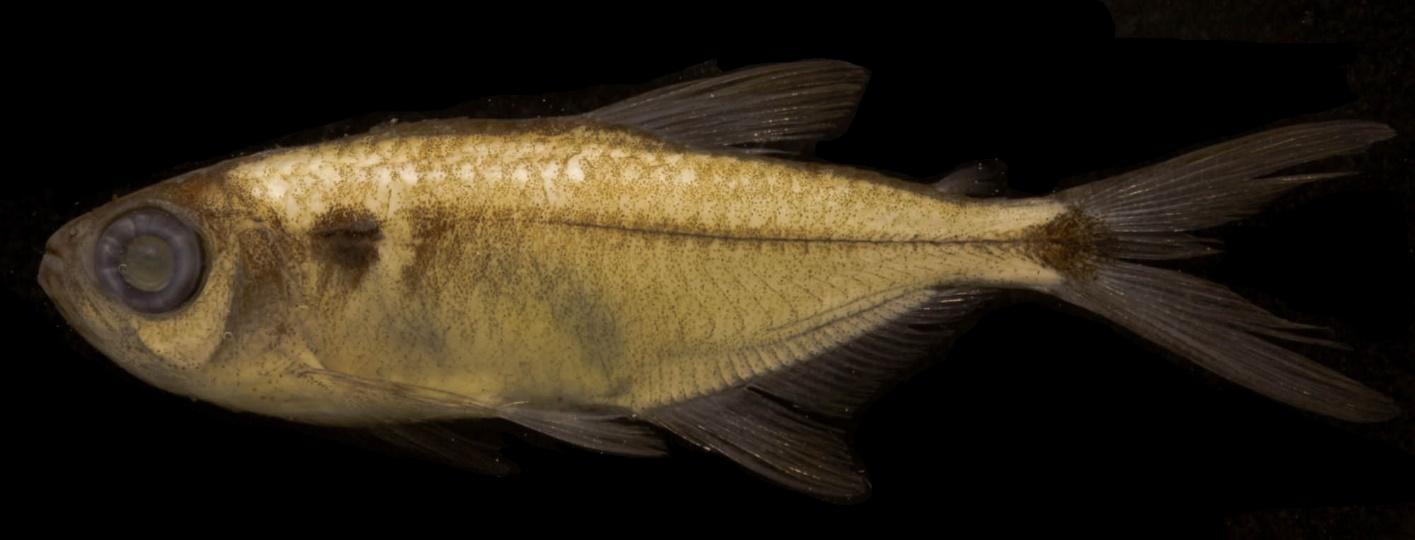


**Figure S14.** *Hemigrammus luelingi*, preserved specimen, MPUJ 14545, 30 mm SL, Caño Danta tributary to Vaupés River at Resguardo de Villa Fátima.


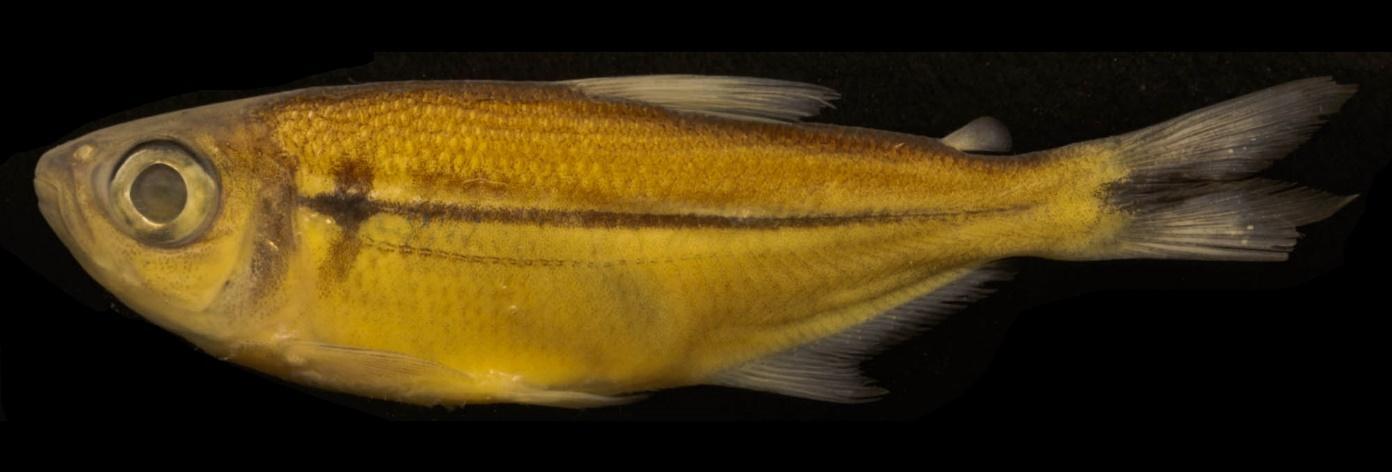


**Figure S15.** *Jupiaba anteroides*, preserved specimen, MPUJ 14487, 55 mm SL, Caño Colibrí at resguardo de Matapí.


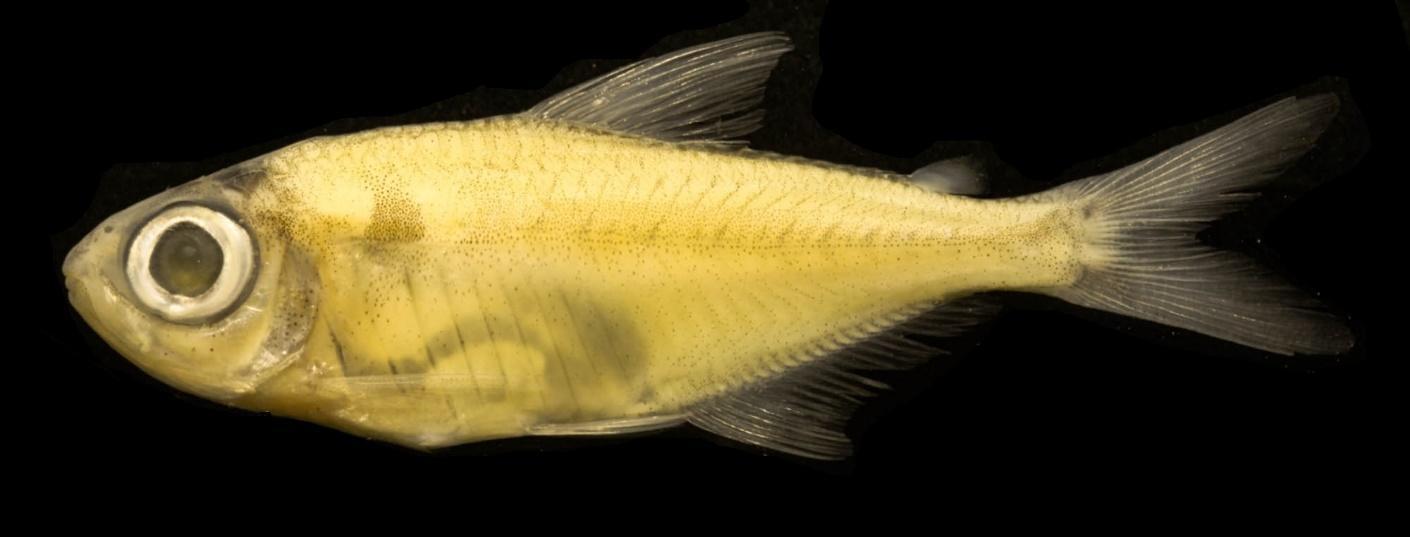


**Figure S16.** *Jupiaba scologaster*, preserved specimen, MPUJ 14436, 37 mm SL, Vaupés River at Resguardo de Macucú.


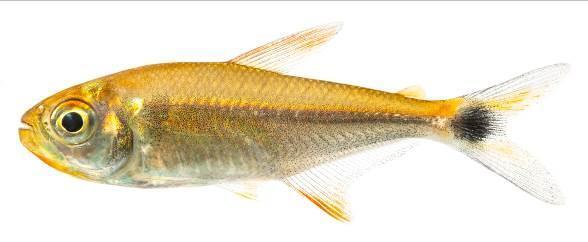


**Figure S17**. *Jupiaba zonata*, live specimen, MPUJ 14435, 53 mm SL, caño Danta tributary Vaupés River, at Resguardo Villa Fátima.


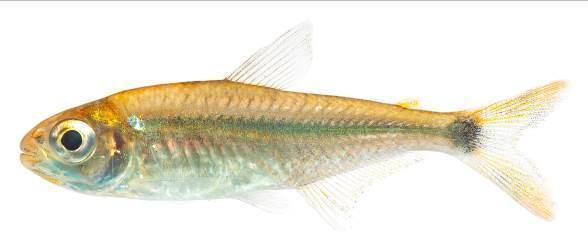


**Figure 4A**. *Jupiaba* sp., live specimen, MPUJ 14475, 48 mm SL Vaupés River Vaupés River at Resguardo de Matapí.


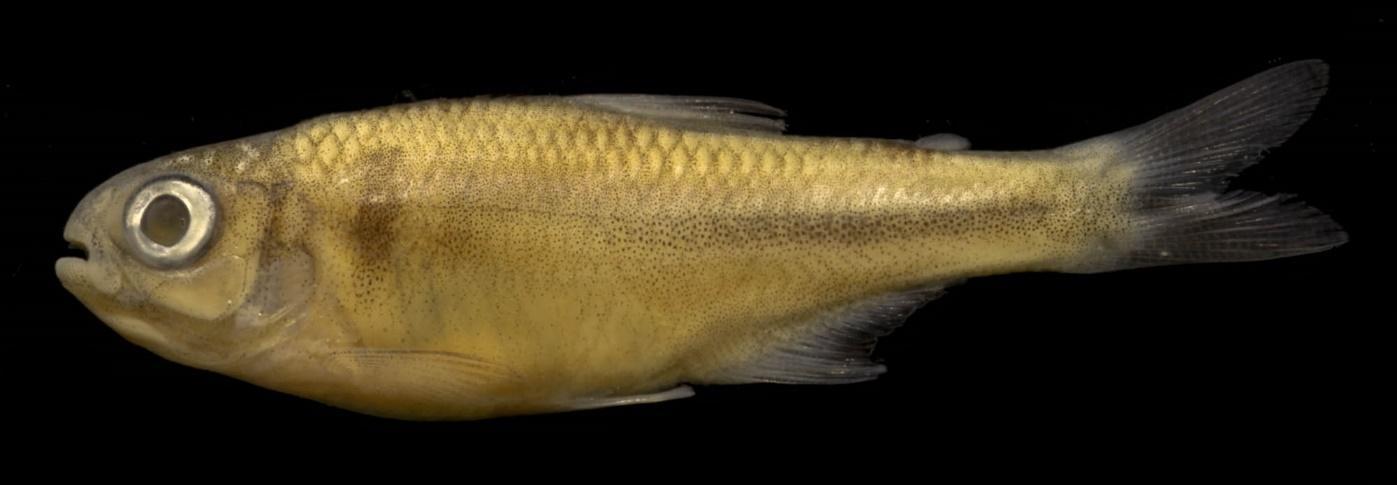


**Figure S19.** *Knodus* sp. 1, preserved specimen, MPUJ 14447, 34 mm SL, Vaupés River sandy beach at Resguardo de Matapí, upstream cachivera Tapira-llerao.


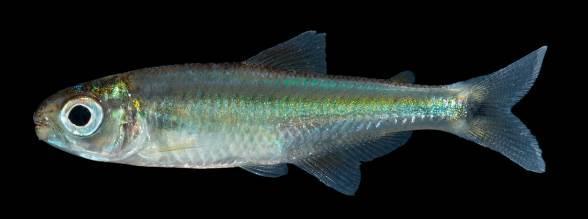


**Figure S20**. *Knodus* sp. 2, live specimen, MPUJ 14536, 38 mm SL, Vaupés River at Resguardo de Villa Fátima.


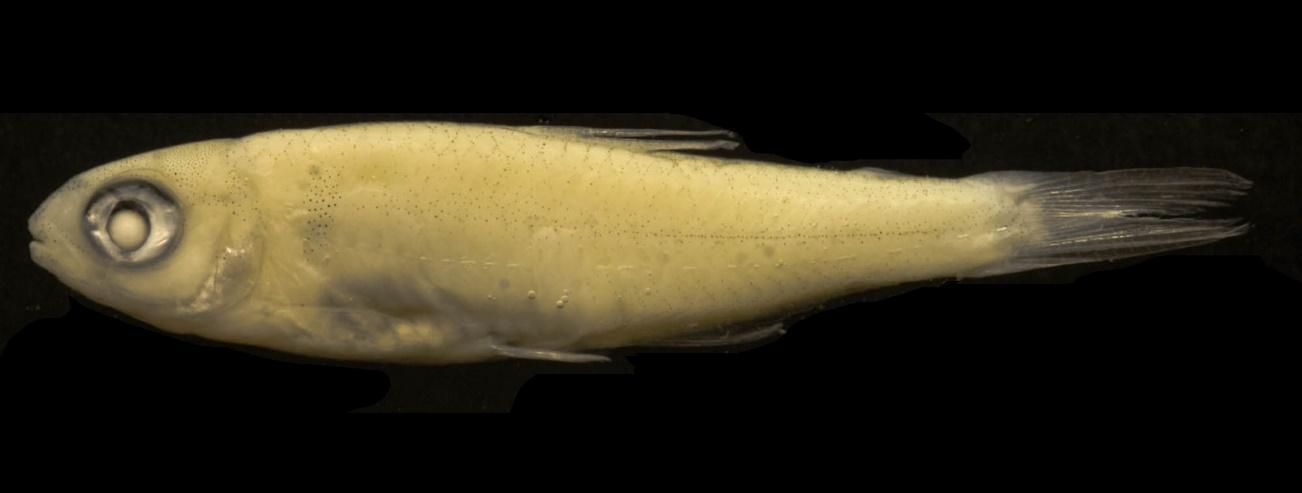


**Figure S21.** *Knodus* sp. 3, preserved specimen, MPUJ 14452, 21 mm SL, Vaupés River at Resguardo de Matapí.


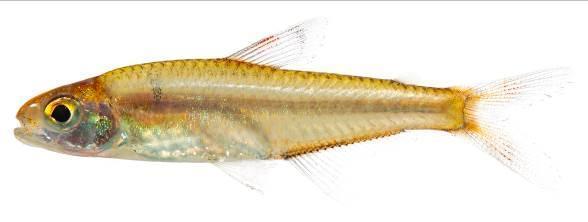


**Figure S22**. *Microschemobrycon callops*, live specimen, MPUJ 14533, 42 mm SL, Vaupés River at Resguardo de Villa Fátima.


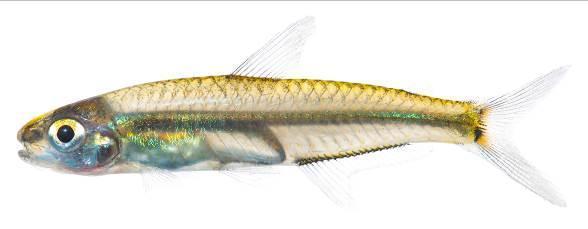


**Figure S23**. *Microschemobrycon casiquiare*, live specimens, MPUJ 14395, 35 mm SL, Vaupés River at Resguardo de Villa Fátima.

**
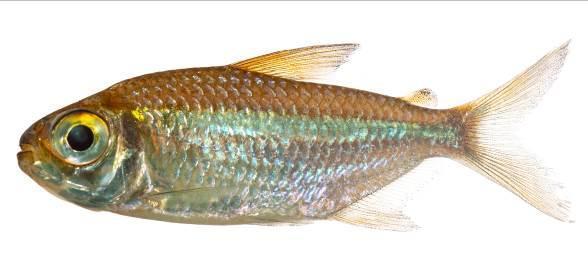
**

**Figure S24**. *Moenkhausia browni*, preserved specimen, MPUJ 16517, 53 mm SL, Vaupés River at Resguardo de Naná.


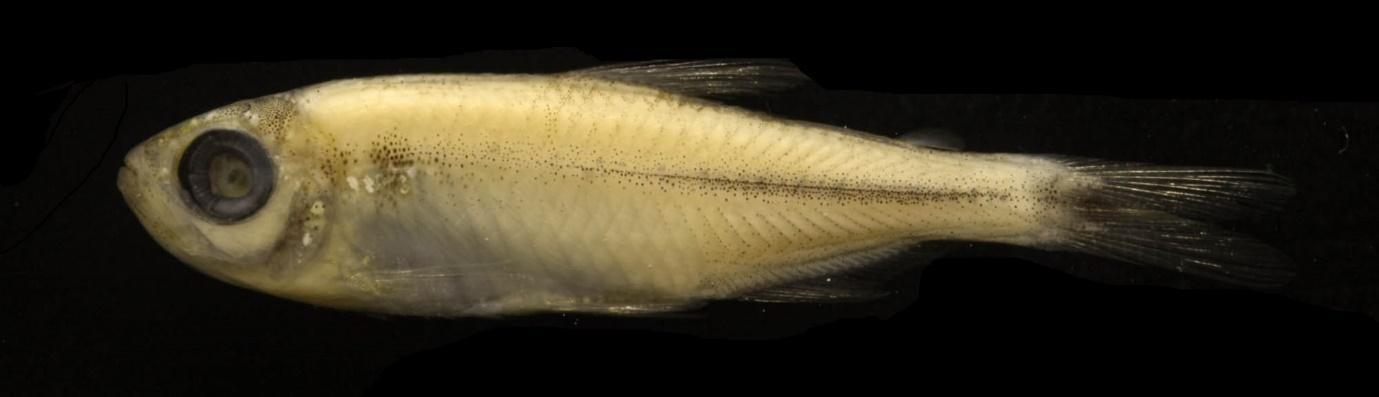


**Figure S25.** *Moenkhausia ceros,* preserved specimen, MPUJ 14366, 32 mm SL, Vaupés River at Resguardo de Trubón.


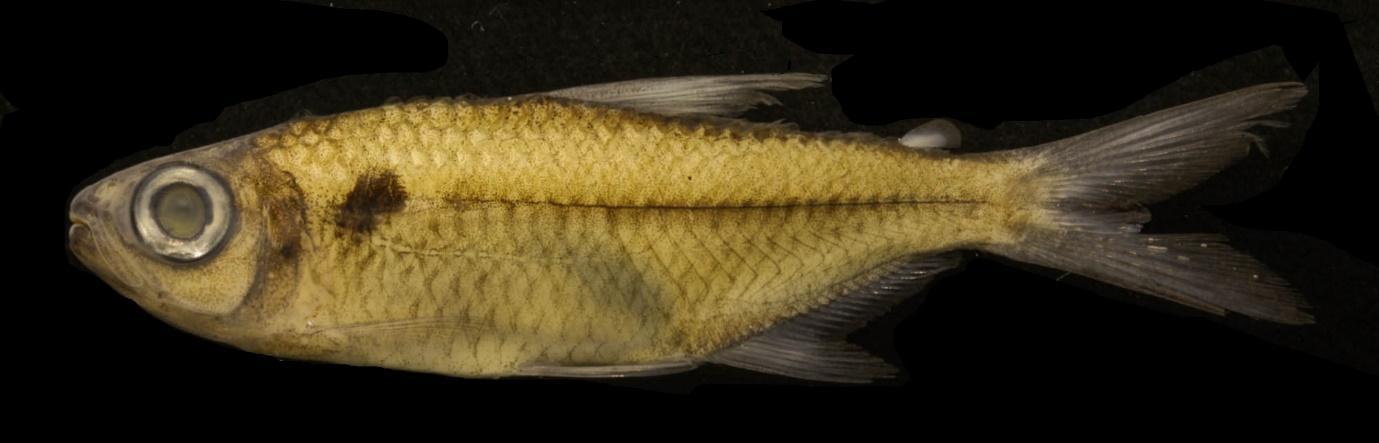


**Figure S26.** *Moenkhausia colletti*, preserved specimen, MPUJ 14460, 38 mm SL, creek tributary to Vaupés River at Resguardo de Matapí.


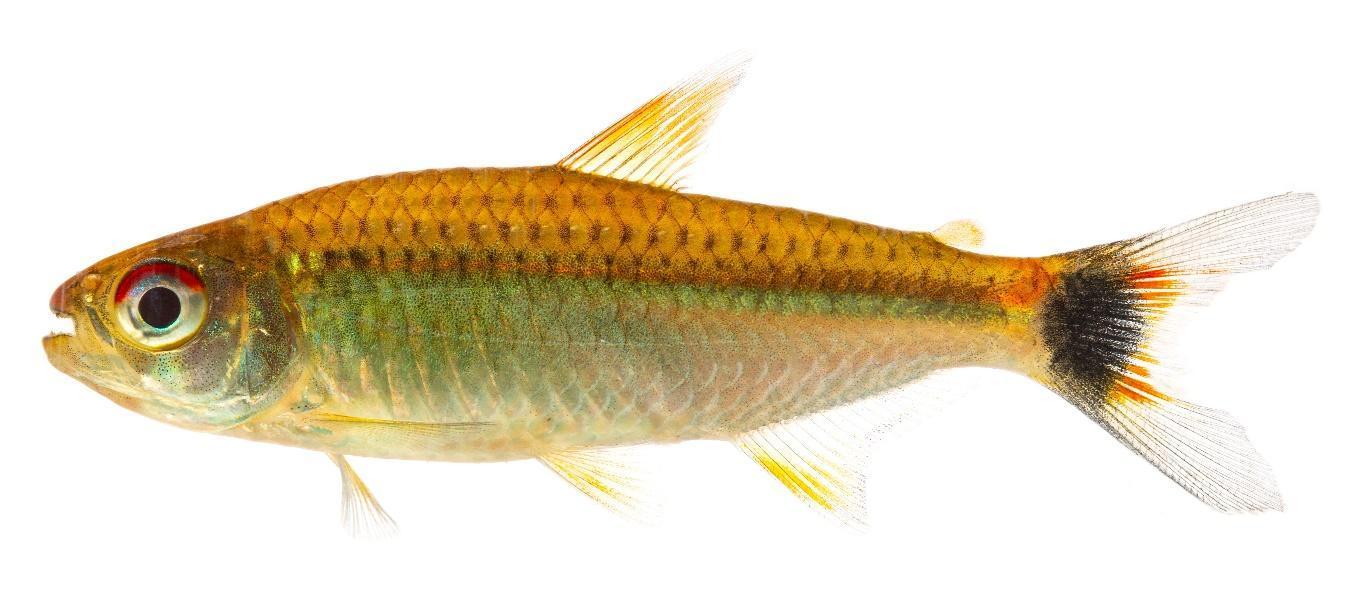


**Figure S27.** *Moenkhausia cotinho,* live specimen, MPUJ 14494, 47 mm SL, Caño Colibrí tributary to Vaupés River at Resguardo de Matapí.


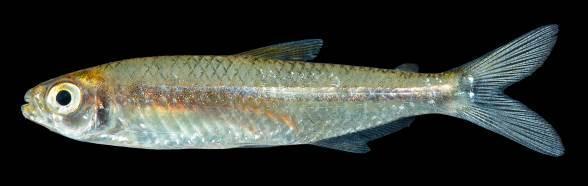


**Figure 4C**. *Moenkhausia* sp., live specimen, MPUJ 14374, 53 mm SL, Vaupés River at Resguardo de Trubón


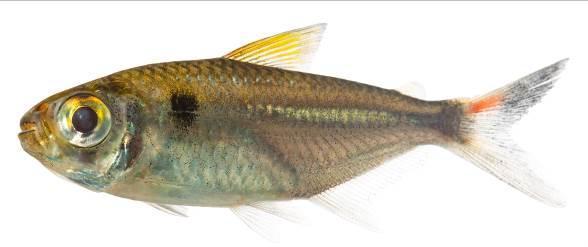


**Figure S29.** *Moenkhausia lata,* live specimen, MPUJ 14432, 46 mm SL, Vaupés River at Resguardo de Macucú.


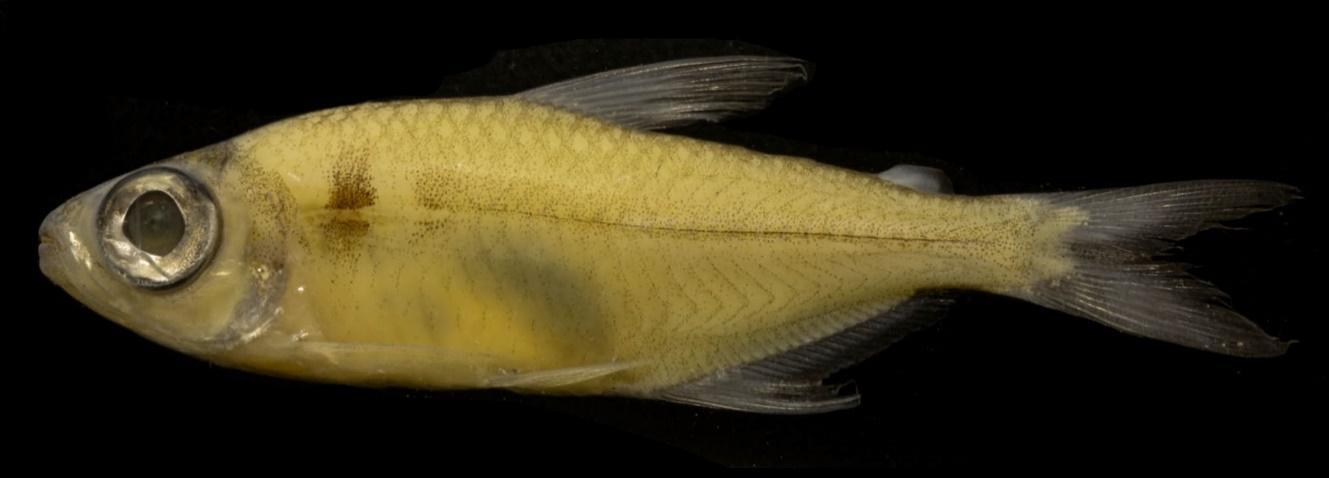


Figure S30. *Moenkhausia melograma,* preserved specimen, MPUJ 14437, 43 mm SL, Vaupés River at Resguardo Macucú.


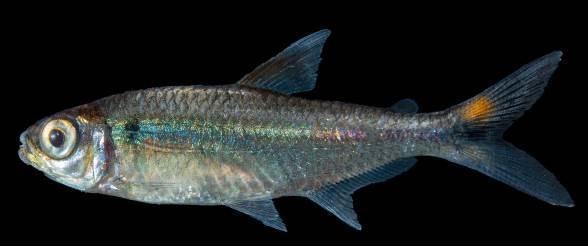


**Figure S31**. *Moenkhausia mikia*, live specimen, MPUJ 14439, 45 mm SL, Vaupés River at Resguardo Macucú.

**
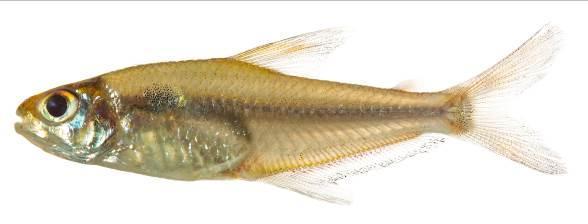
**

**Figure S32.** *Phenacogaster* sp. 1, live specimen, MPUJ 14373, 38 mm SL, Vaupés River at Resguardo Trubón.


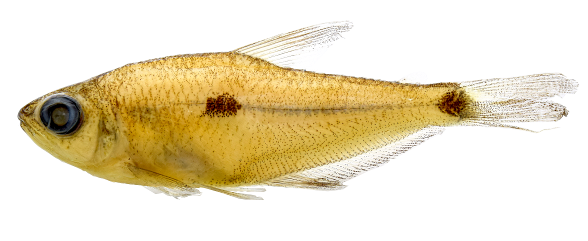


**Figure 4B.** *Phenacogaster* sp. 2, preserved specimen, MPUJ 14364, 34.9 mm SL, Vaupés River at Resguardo Trubón.


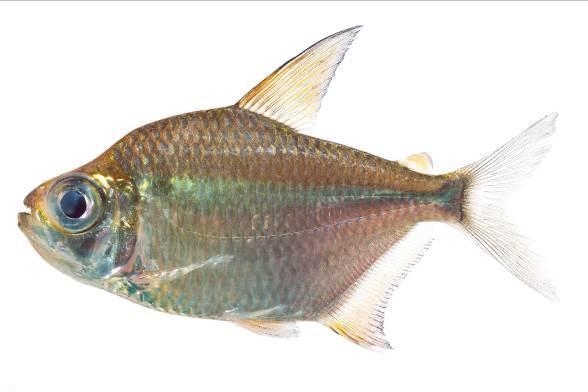


**Figure S34.** *Tetragonopterus chalceus*, live specimen, MPUJ 14483, 65.4 mm SL, Caño Colibrí at Resguardo de Matapí.


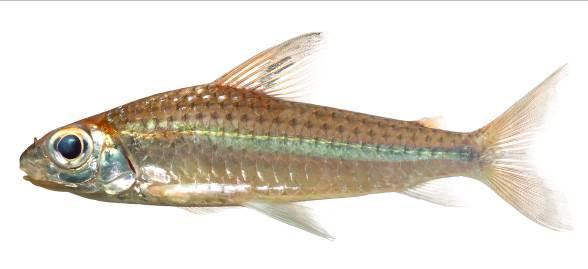


**Figure S35**. *Caenotropus labyrinthicus*, live specimen, MPUJ 16516, 95 mm SL, Vaupés River at Resguardo de Macucú.


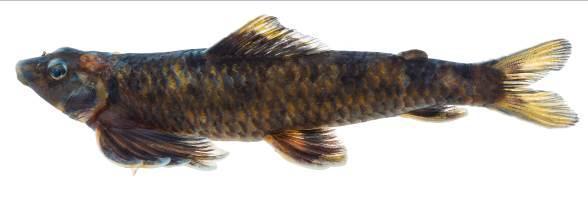


**Figure S36.** *Characidium declivirostre*, live specimen, MPUJ 14497, 72.9 mm SL, Vaupés River at Resguardo de Matapí.


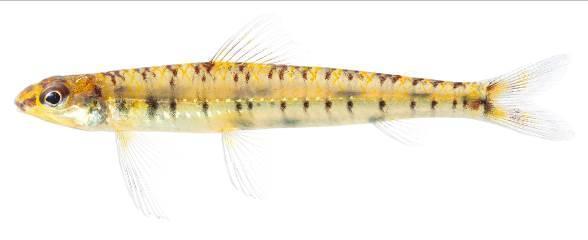


**Figure S37.** *Characidium longum*, live specimen, MPUJ 14365, 37.9 mm SL, Vaupés River at Resguardo Trubón.


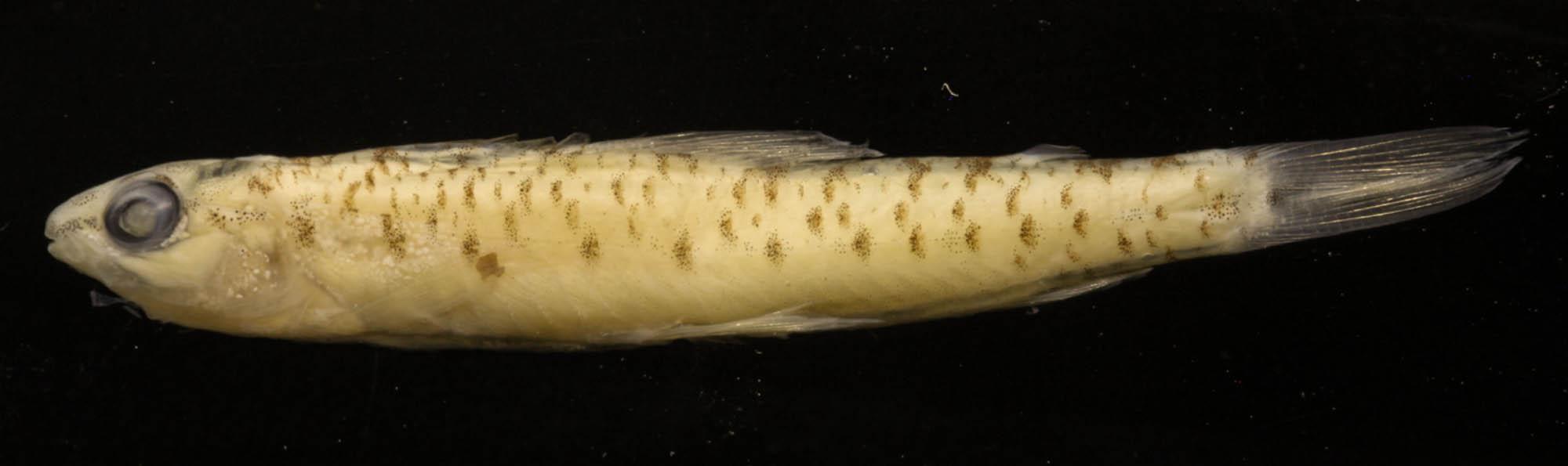


**Figure S38.** *Characidium pteroides*, preserved specimen, MPUJ 14384, 29.7 mm SL, Vaupés River at Resguardo de Villa Fátima.


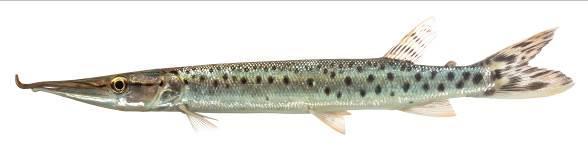


**Figure S39.** *Boulengerella maculata*, live specimen, MPUJ 14502, 192 mm SL, Vaupés River at Resguardo de Macucú.


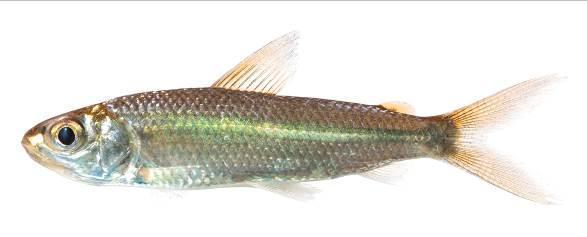


**Figure S40.** *Cyphocharax leucostictus*, live specimen, MPUJ 14418, 80 mm SL, Vaupés River at Resguardo de Villa Fátima.


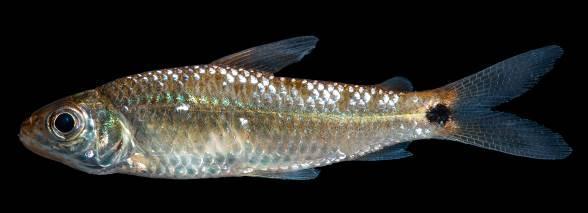


**Figure S41.** *Cyphocharax spilurus*, live specimen, MPUJ 14391, 35.6 mm SL, Vaupés River at Resguardo de Villa Fátima.


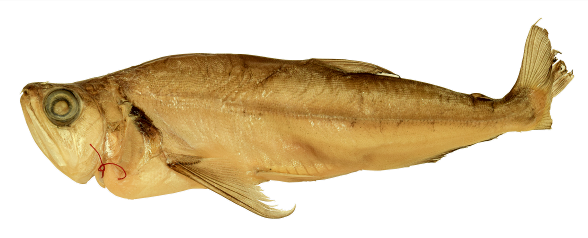


**Figure S42.** *Hydrolicus wallacei*, preserved specimen, MPUJ 14547, 191 mm SL, Vaupés River at Resguardo de Villa Fátima.

**
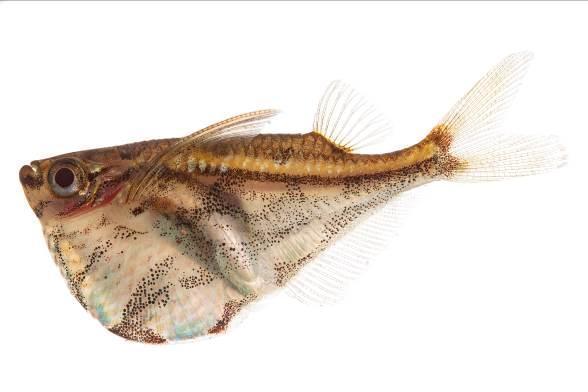
**

**Figure S43.** *Carnegiella strigata*, live specimen, MPUJ 14493, 26.7 mm SL, Caño Colibrí at Resguardo de Matapí.

**
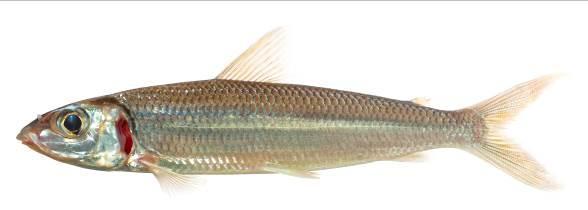
**

**Figure S44.** *Argonectes longiceps*, live specimen, MPUJ 16519, 120 mm SL, Vaupés River at Resguardo de Macucú.

**
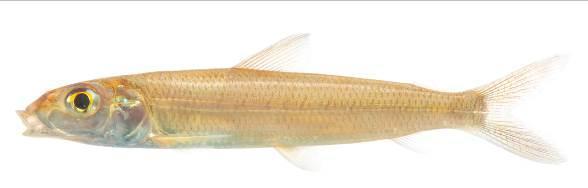
**

**Figure S45**. *Bivibranchia fowleri,* live specimen, MPUJ uncatalogued mm SL, Vaupés River at Resguardo de Trubón.


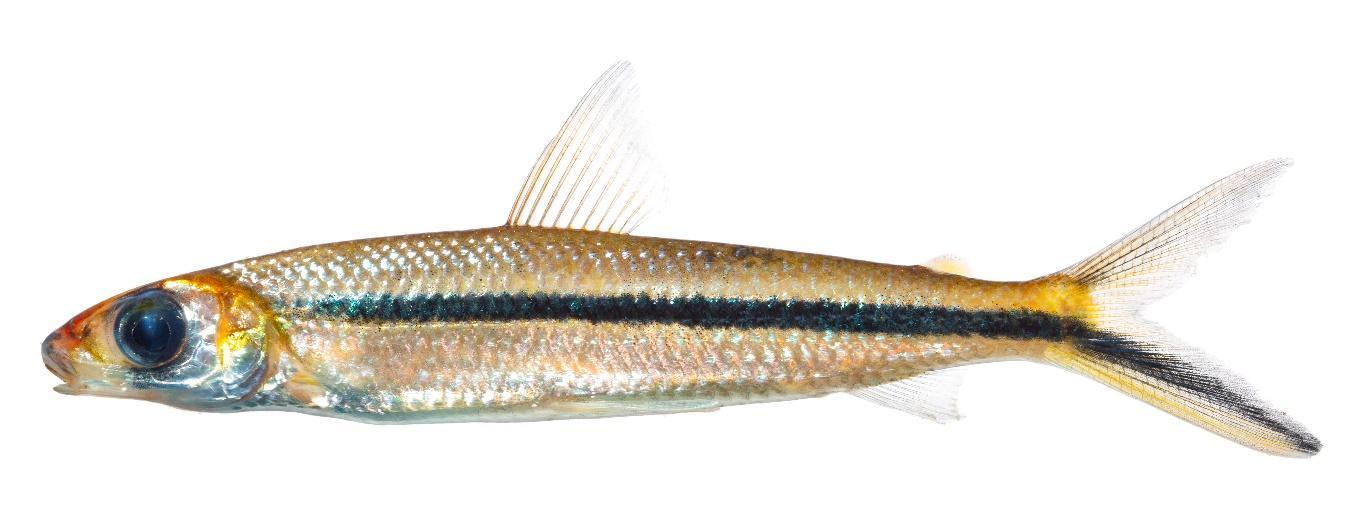


**Figure S46.** *Hemiodus thayeria* live specimen, MPUJ 14514, 100.5 mm SL, Vaupés River at Resguardo de Villa Fátima.

**
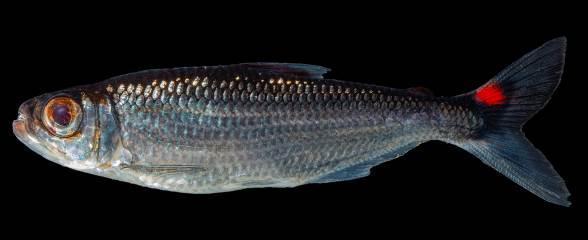
**

**Figure S47**. *Bryconops giacopinii* live specimen, MPUJ 14462, 65.4 mm SL, creek tributary to Vaupés River at Resguardo de Matapí.

**
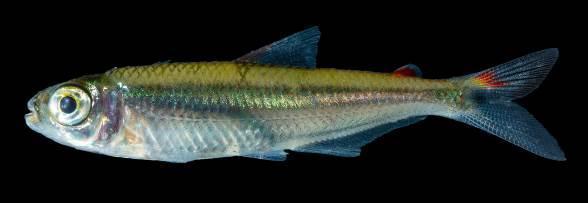
**

**Figure S48**. *Bryconops collettii* live specimen, MPUJ 14461, 53.1 mm SL, creek tributary to Vaupés River at Resguardo de Matapí.

**
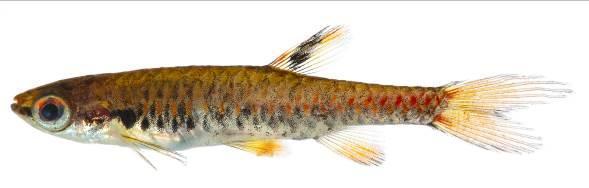
**

**Figure S49.** *Copella nattereri*, live specimen, MPUJ  14548, 32 mm SL, Caño Danta tributary to Vaupés River at Resguardo de Villa Fátima.

**
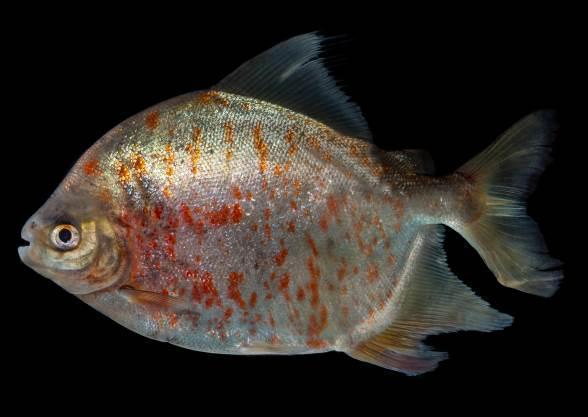
**

**Figure 3A.** *Myloplus lucienae*, live specimen, MPUJ 14528, 288 mm SL, Vaupés River at Resguardo de Macucú.

**
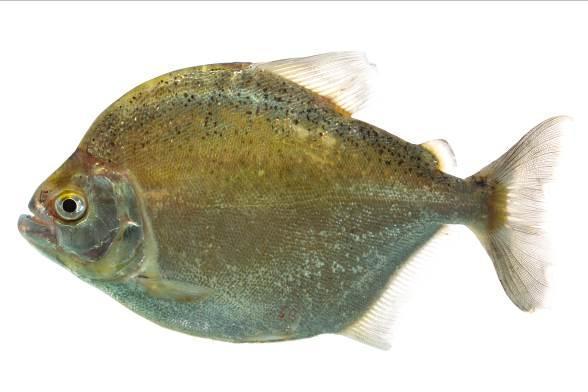
**

**Figure S51.** *Serrasalmus* *striolatus*, live specimen, MPUJ uncatalogued Vaupés River at Resguardo Trubón.
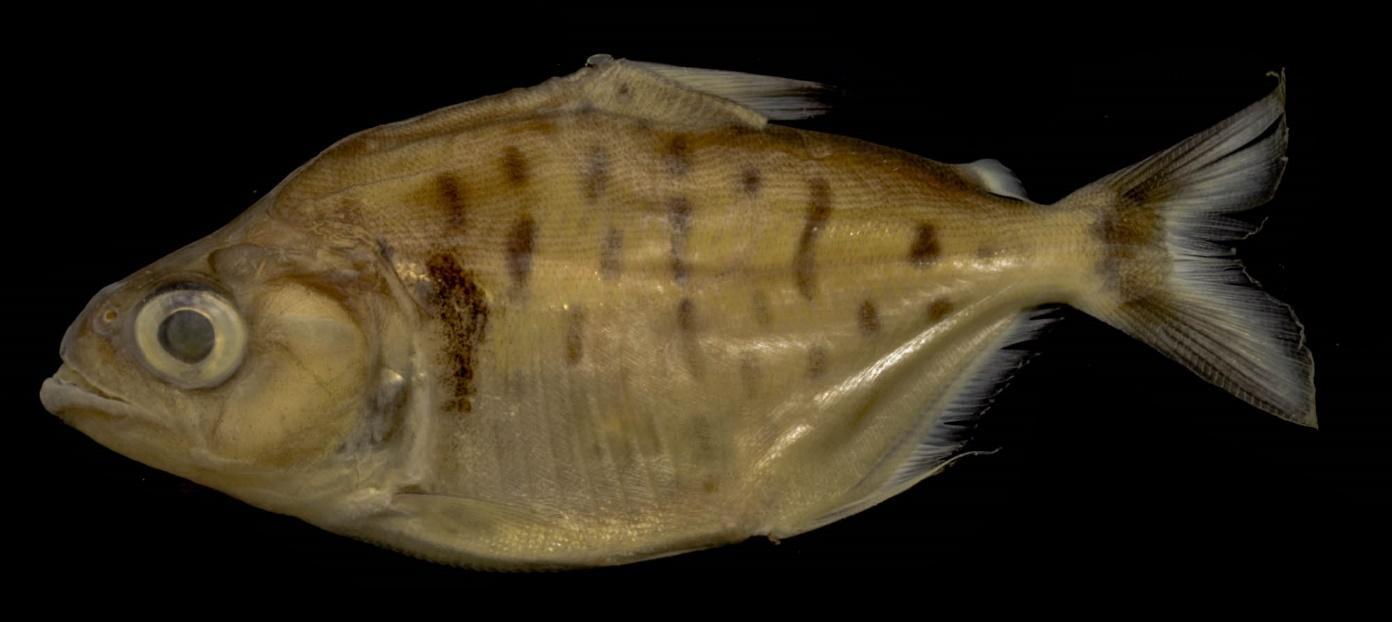


**Figure S52.** *Serrasalmus manueli*, preserved specimen, MPUJ 14417, 93 mm SL, Vaupés River at Resguardo de Macucú.


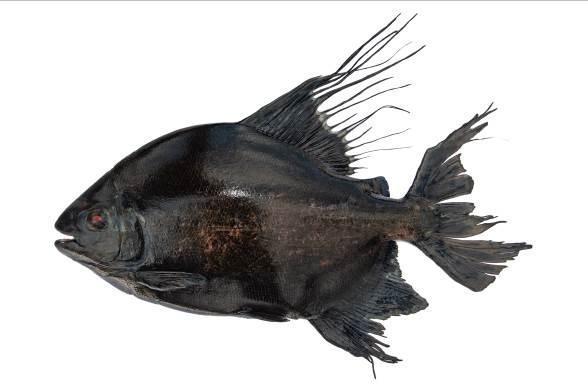


**Figure 3B.** *Tometes makue*, live specimen, MPUJ uncatalogued, 380 mm SL, Vaupés River at Resguardo Trubón.


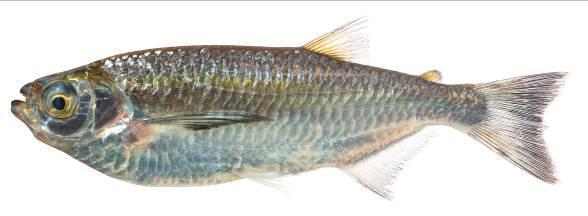


**Figure S54.** *Triportheus albus*, live specimen, MPUJ 16522, 120.2 mm SL, Vaupés River at Resguardo de Macucú.

**
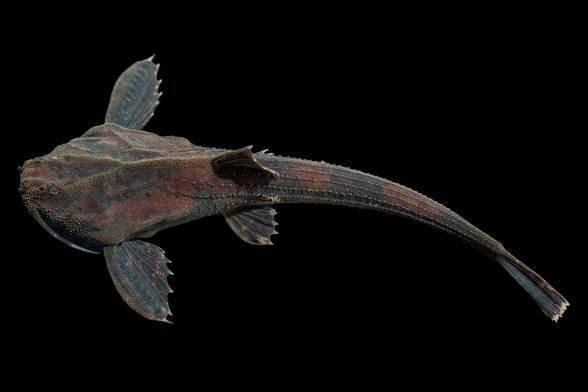
**

**Figure 4D.** *Bunocephalus* sp., live specimen, MPUJ 14433, 54 mm SL, Vaupés River at Resguardo de Macucú.

**
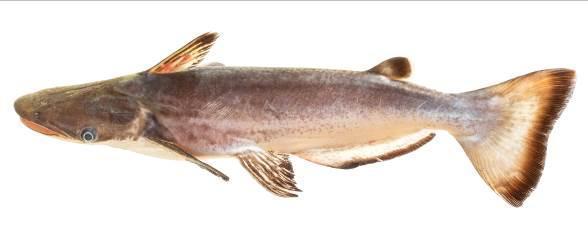
**

**Figure S56.** *Ageneiousus inermis*, live specimen, MPUJ 14515, 160 mm SL, Vaupés River at Resguardo de Macucú.

**
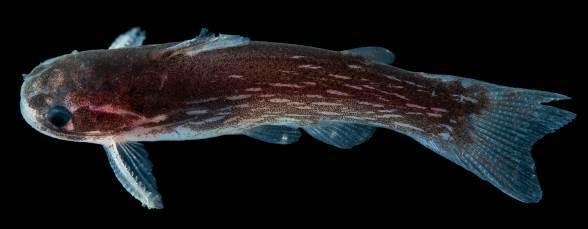
**

**Figure S57**. *Tatia intermedia*, live specimen, MPUJ uncatalogued, 51 mm SL, Vaupés River Resguardo de Trubón.

**
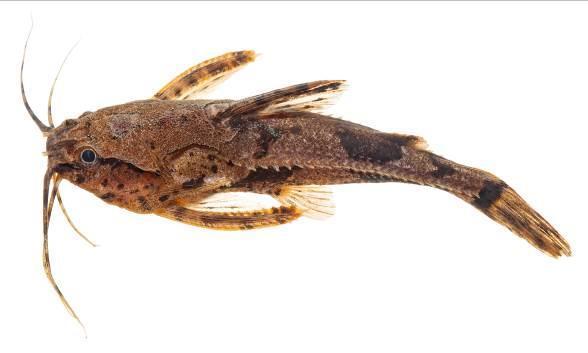
**

**Figure S58.** *Amblydoras affinis*, live specimen, MPUJ 14398, 64.3 mm SL Vaupés River at Resguardo de Villa Fátima.

**
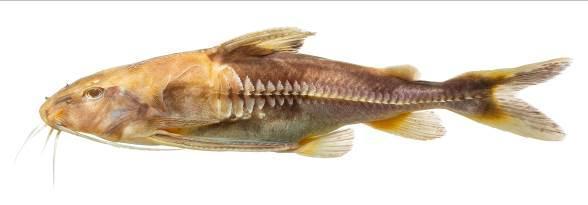
**

**Figure S59**. *Centrodoras hasemani*, live specimen, MPUJ uncatalogued, 250 mm SL, Vaupés River at Resguardo de Trubón.

**
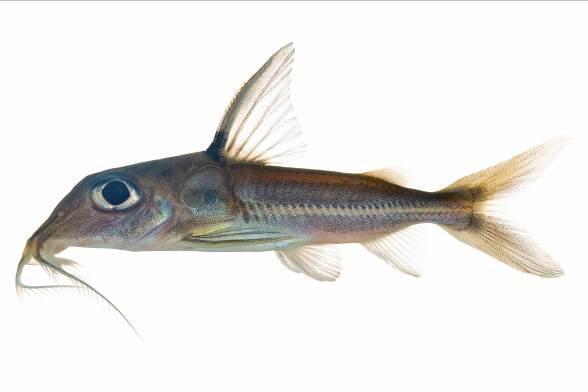
**

**Figure S60.** *Doras phlyzakion*, live specimen, MPUJ uncatalogued, 110 mm SL, Vaupés River at Resguardo de Macucú.

**
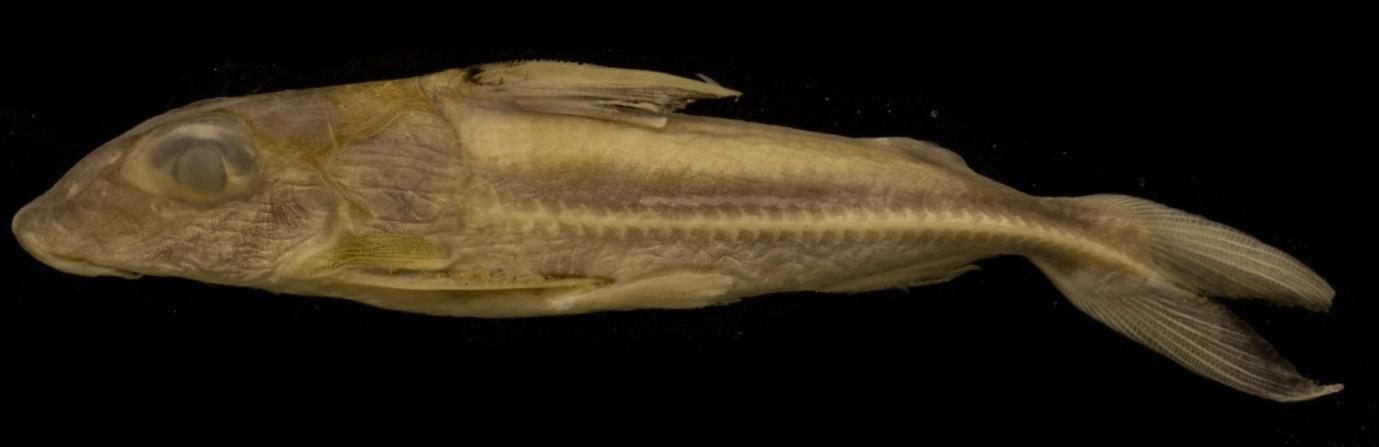
**

**Figure S61-Fig. 3C** *Leptodoras praelongus*, preserved specimen, MPUJ 16518, 95.2 mm SL, Vaupés River at Resguardo de Macucú.

**
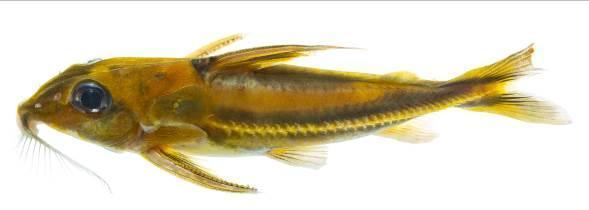
**

**Figure S62**. *Tenellus ternetzi*, live specimen, MPUJ uncatalogued, 132 mm SL, Vaupés River at Resguardo Trubón.

**
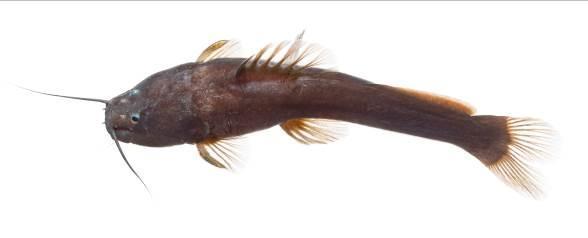
**

**Figure S63.** *Leptoglanis nocturna*, live specimen, MPUJ Uncatalogued, 65 mm SL, Vaupés River at Resguardo de Trubón.


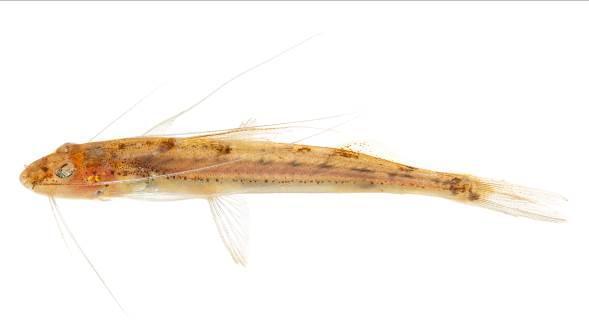


**Figure S64**. *Mastiglanis asopos*, live specimen, MPUJ 14401, 63.2 mm SL, Vaupés River at Resguardo de Villa Fátima.

**
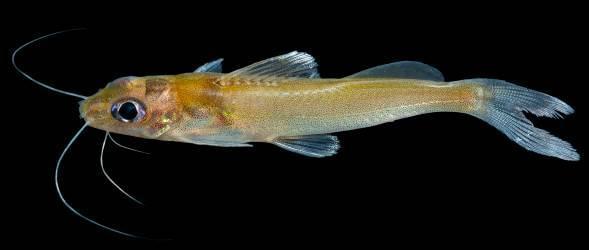
**

**Figure S65.** *Pimelodella* sp., live specimen, MPUJ 14402, 70.5 mm SL, Vaupés River at Resguardo de Villa Fátima.

**
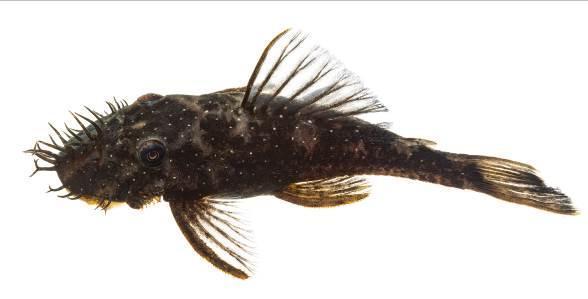
**

**Figure S66.** *Ancistrus patronus*, live specimen, MPUJ 14482, 61.2 mm SL, small lagoon isolated from Vaupés River near Resguardo de Matapí.

**
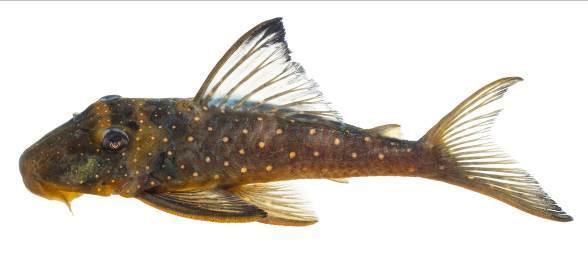
**

**Figure 4F.** *Hemiancistrus* sp., live specimen, MPUJ 14520, 90.5 mm SL. Vaupés River at Resguardo de Matapí.

**
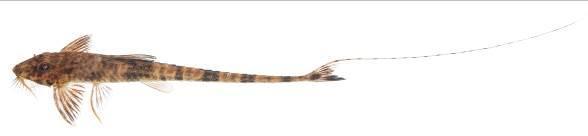
**

**Figure S68.** *Loricaria cataphracta*, live specimen, MPUJ 14401, 170 mm SL, Vaupés River at Resguardo de Villa Fátima.

**
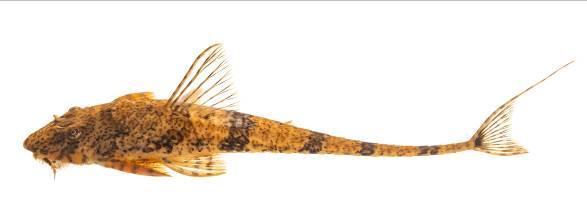
**

**Figure S69***. Rineloricaria* sp. 1, live specimen, MPUJ 14530, 12.5 mm SL, Vaupés River at Resguardo de Villa Fátima.

**
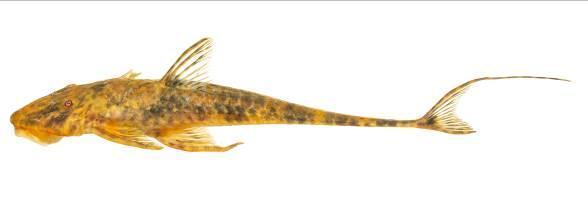
**

**Figure S70**. *Rineloricaria cachivera* live specimen, MPUJ uncatalogued, Vaupés River at Resguardo Trubón.


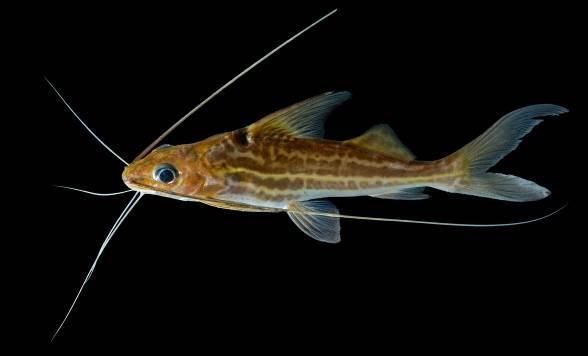


**Figure S71***. Pimelodus albofasciatus*, live specimen, MPUJ 14503, 12.5 mm SL, Vaupés River at Resguardo de Matapí.

**
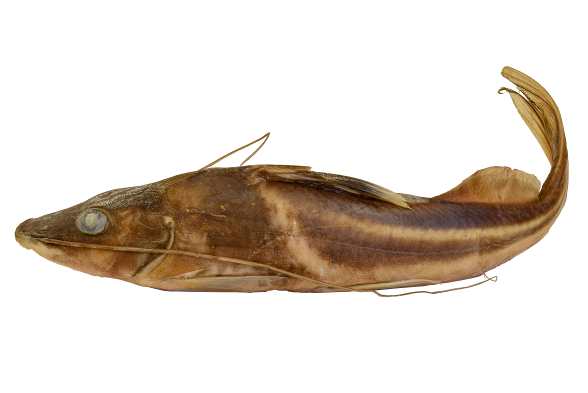
**

**Figure S72.** *Pimelodus ornatus*, preserved specimen, MPUJ 14518, 242 mm SL, Vaupés River at Resguardo de Villa Fátima.

**
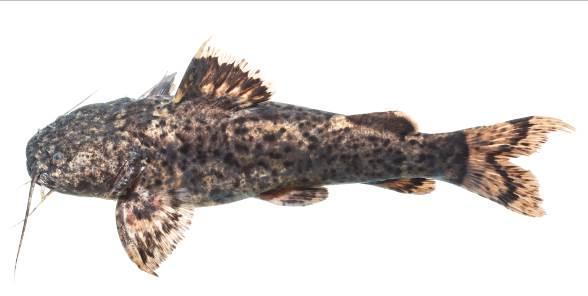
**

**Figure S73**. *Pseudopimelodus bufonius*, live specimen, MPUJ uncatalogued, 111,5 mm SL Vaupés River at Resguardo Trubón.

**
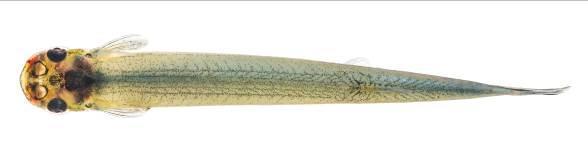
**

**Figure S74.** *Haemomaster venezuelae*, live specimen, MPUJ 14395, 50.5 mm SL, Vaupés River at Resguardo de Villa Fátima.

**
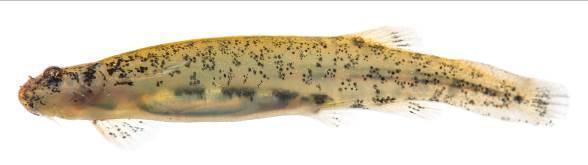
**

**Figure S75**. *Ochmacanthus reinhardti*, live specimen, MPUJ 14387, 45.1 mm SL, Vaupés River at Resguardo de Villa Fátima.

**
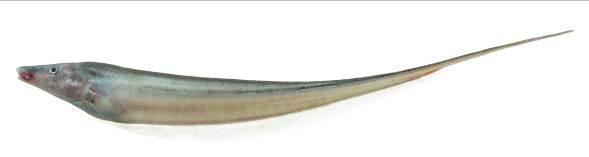
**

**Figure 4E.** *Archolaemus* sp., live specimen, MPUJ uncatalogued Vaupés River at Resguardo de Trubón.

**
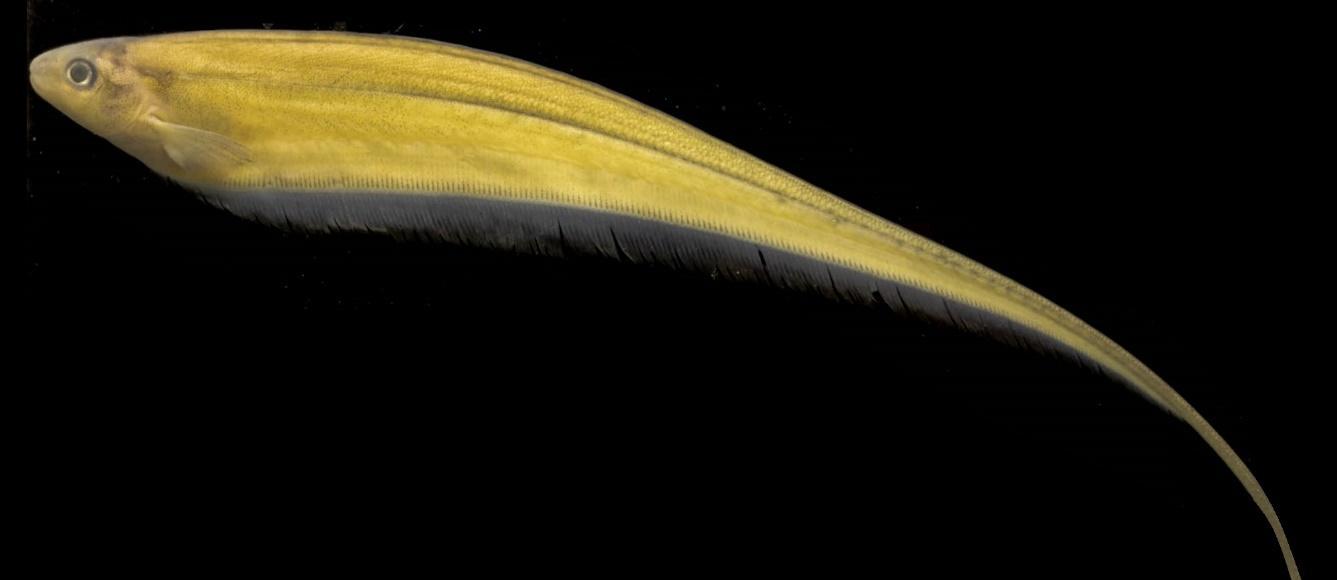
**

**Figure S77-Fig. 3D.** *Eigenmannia matintapereira*, preserved specimen, MPUJ 14501, 120 mm SL, Vaupés River at Resguardo de Macucú.

**
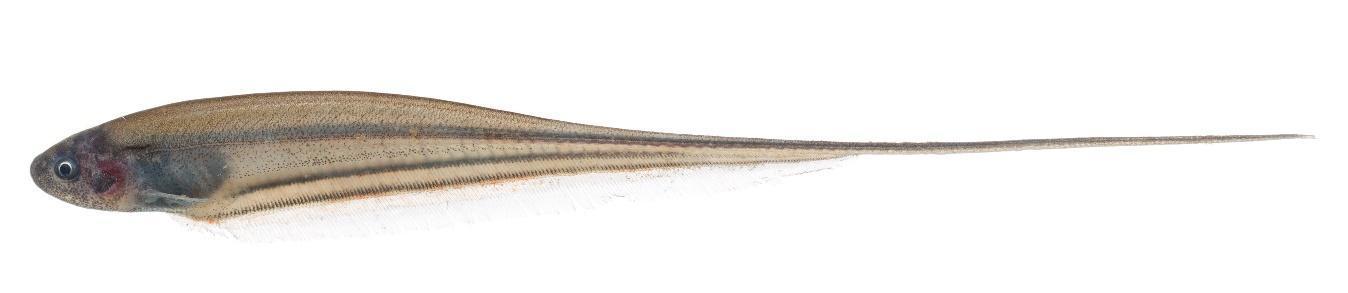
**

**Figure S78**. *Eigenmannia* sp., live specimen, MPUJ 14393, 98 mm SL, Rio Vaupés at Resguardo deVilla Fátima.

**
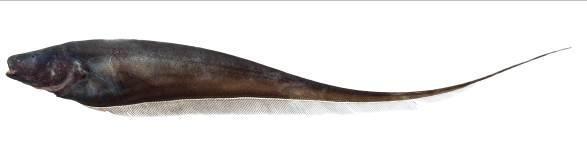
**

**Figure S79**. *Sternopygus macrurus*, live specimen, MPUJ uncatalogued, Vaupés River at Resguardo de Trubón.

**
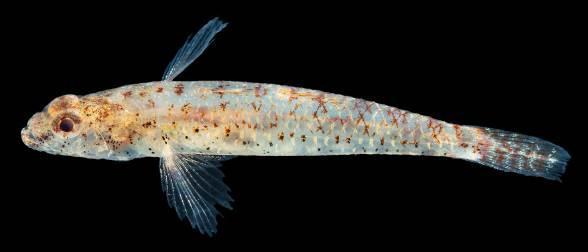
**

**Figure S80.** *Microphilypnus ternetzi*, live specimen, MPUJ 14466, 19. 6 mm SL, Vaupés River at Resguardo de Matapí


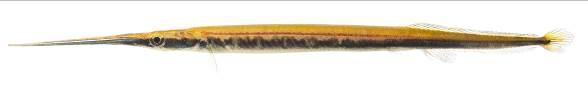


**Figure S81**. *Potamorrhaphis guianensis*, live specimen, MPUJ 14508, 201.8 mm SL, Caño Colibrí at Resguardo de Matapí.

**
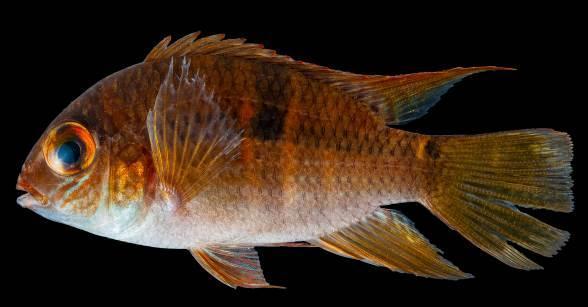
**

**Figure S82**. *Aequidens diadema* sp. live specimen, MPUJ 14552, 70 mm SL, Caño Danta tributary to Vaupés River at Resguardo de Villa Fátima.

**
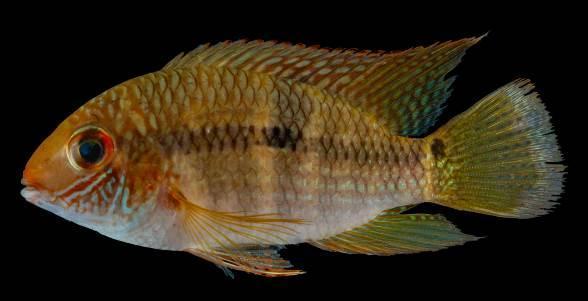
**

Figure S83. *Aequidens tetramerus* live specimen, MPUJ 14459, 75 mm SL, creek tributary to Vaupés River near Resguardo de Matapí.

**
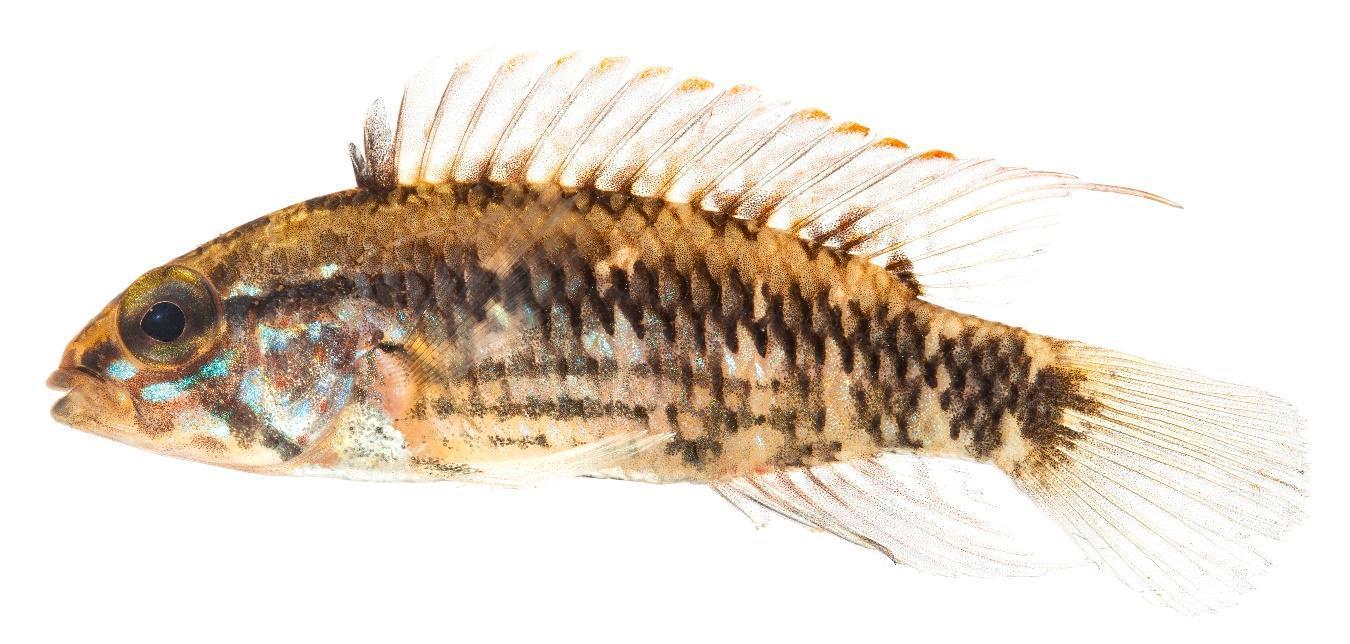
**

**Figure S84.** *Apistogramma* sp. 1, live specimen, MPUJ 14551, 45 mm SL, Vaupés River at Resguardo de Villa Fátima.

**
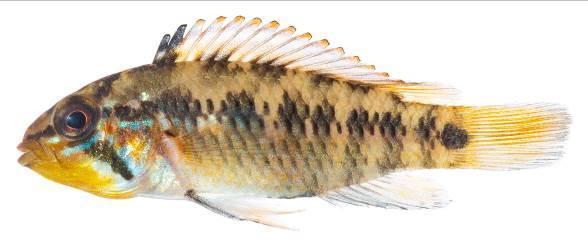
**

**Figure S85***. Apistogramma* sp. 2, live specimen, MPUJ 14549, 47 mm SL, Caño Danta tributary to Vaupés River at Resguardo de Villa Fátima.

**
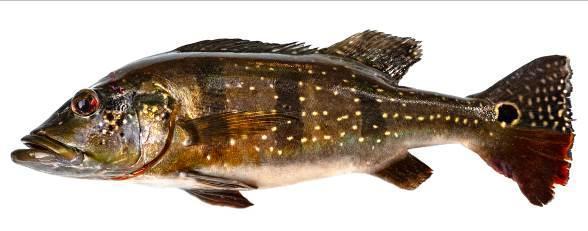
**

**Figure S86.** *Cichla temensis*, specimen just before preservation, MPUJ uncatalogued Vaupés River Resguardo de Matapí.

**
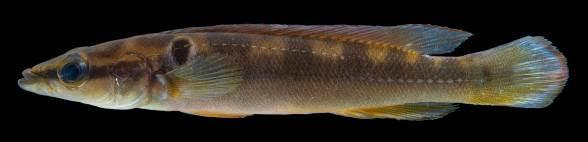
**

**Figure S87.** *Saxatilia alta*, live specimen, MPUJ 14474, 70.5 mm SL, Vaupés River at

Resguardo de Macucú

**
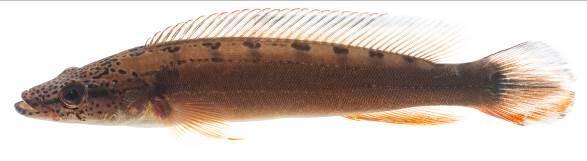
**

**Figure S88.** *Lugubria lenticulata*, live specimen, MPUJ 14505, 141 mm SL, Vaupés River at Resguardo de Matapí.

**
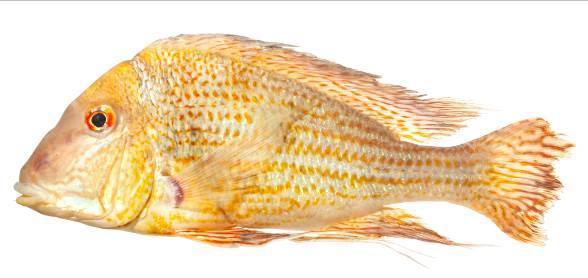
**

**Figure S89.** *Geophagus abalios*, live specimen, MPUJ 14526, 150.5 mm SL, Vaupés River at Resguardo de Macucú

**
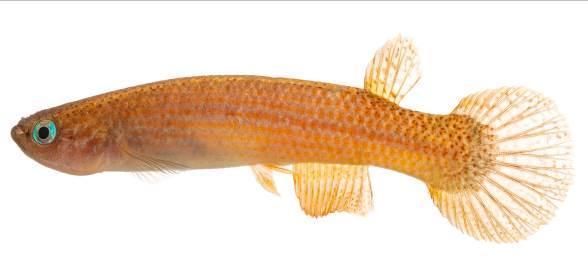
**

**Figure S90**. *Anablepsoides* sp., live specimen, MPUJ 14485, 45.2 mm SL, Caño Colibrí at Resguardo de Matapí.

**
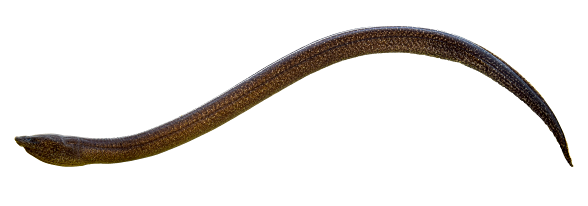
**

**Figure S91.** *Synbranchus marmoratus*, preserved specimen, MPUJ 14500, 112 mm SL,

caño Almidón.

**
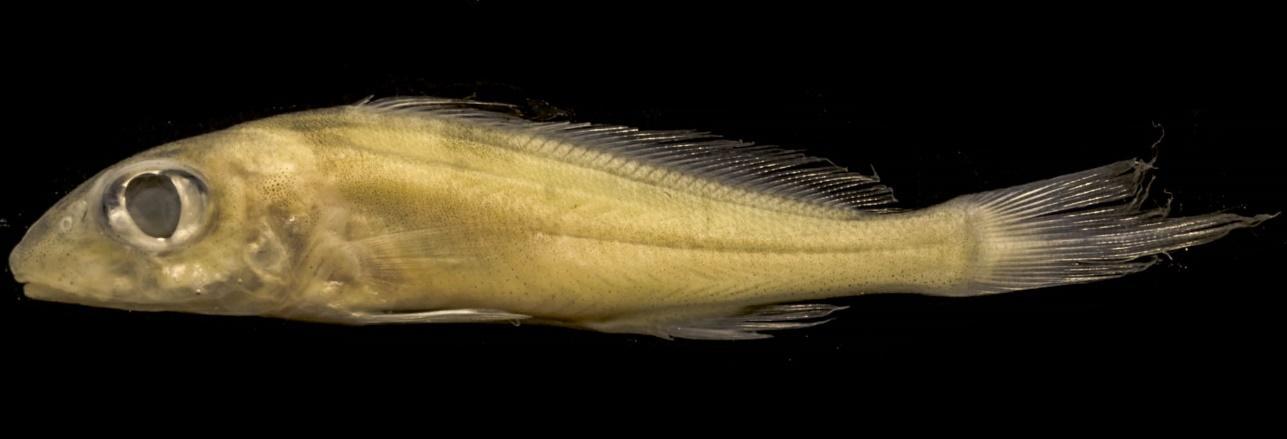
**

**Figure S92.** *Pachyurus gabrielensis*, preserved specimen, MPUJ 14441, 54.5 mm SL, Vaupés River at Resguardo de Macucú.

**
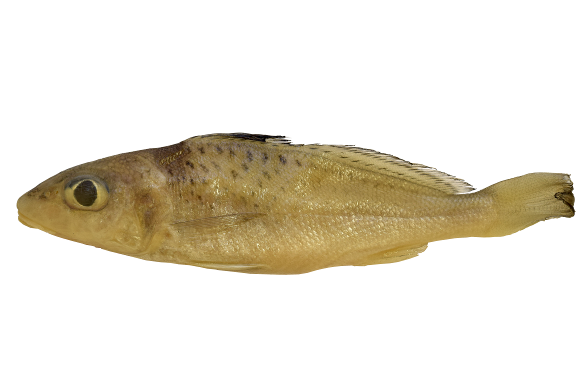
**

**Figure S93.** *Pachyurus junki*, preserved specimen, MPUJ 14511, 159 mm SL, Vaupés River at Resguardo de Macucú.

**
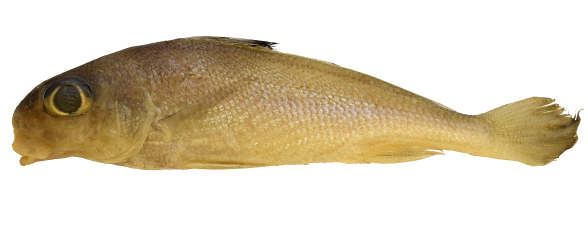
**

**Figure S94.** *Pachyurus schomburgkii*, preserved specimen, MPUJ 14512, 151 mm SL, Vaupés River at Resguardo de Macucú.

**
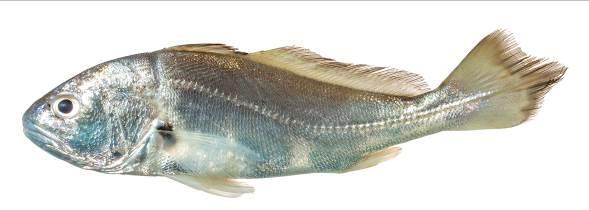
**

**Figure S95.** *Plagioscion squamosissimus*, live specimen, MPUJ uncatalogued Vaupés River.
